# A Theoretical Study on Reductive Debromination of Polybrominated Diphenyl Ethers

**DOI:** 10.3390/ijms13079332

**Published:** 2012-07-24

**Authors:** Ji-Wei Hu, Yuan Zhuang, Jin Luo, Xiong-Hui Wei, Xian-Fei Huang

**Affiliations:** 1 Guizhou Provincial Key Laboratory for Information System of Mountainous Areas and Protection of Ecological Environment, Guizhou Normal University, Guiyang 550001, China; E-Mails: luojin@gznu.edu.cn (J.L.); hxfswjs@gznu.edu.cn (X.-F.H.); 2 Department of Civil and Environmental Engineering, Stanford University, Stanford, CA 94305, USA; E-Mail: zhuangy@stanford.edu; 3 Department of Applied Chemistry, College of Chemistry and Molecular Engineering, Peking University, Beijing 100871, China; E-Mail: xhwei@pku.edu.cn

**Keywords:** polybrominated diphenyl ethers, reductive debromination, radical anion, density functional theory, electron transfer

## Abstract

Recent progress has been made in the reductive debromination of polybrominated diphenyl ethers (PBDEs) by nanoscale zero-valent iron (nZVI). To better understand the mechanism of this reaction, seven selected BDE congeners and their anions were investigated at the density functional theory (DFT) level using four different methods, including B3LYP/6-31G(d), B3LYP/6-31+G(d), B3LYP/6-31G(d,p) and B3LYP/6-311G(d,p). The cleaved C–Br bonds observed in the equilibrium structures of anionic PBDEs were adopted as the probe of the susceptible debromination position of PBDEs in the presence of nZVI, and the proposed major reaction pathways based on our calculations can satisfactorily conform to the reported experimental results. The debromination preference is theoretically evaluated as meta-Br > ortho-Br > para-Br. In addition, both the calculated frontier orbital energies and adiabatic electronic affinities were found to be highly related to their experimental reductive debromination rate constants. The highest linear regression coefficient was observed in the case using the energy of lowest unoccupied molecular orbital as the molecular descriptor obtained from B3LYP/6-31G(d) (*R*^2^ = 0.961, *n* = 7) or B3LYP/6-31G(d,p) (*R*^2^ = 0.961, *n* = 7). The results clearly showed the evidence of an electron transfer mechanism associated with this reductive debromination reaction.

## 1. Introduction

Polybrominated diphenyl ethers (PBDEs) have been widely used as additive flame retardants in polymers in many consumer products. During the manufacture, disposal, dismantling and recycling of plastics, foam and other PBDE-containing articles, PBDEs could be released into the environment and would end up in air, water, soil, sediment and the food chain, where they can accumulate in wildlife and in people [1,2]. They are generally persistent or semi-persistent, and bioaccumulative in the environment and could pose emerging risks to humans via effects such as endocrine disruption [2–6]. Although the ban of specific lowly brominated congeners of PBDEs has been adopted in some regions [7–9], continued use, degradation and biotransformation of deca-BDE will still lead to the accumulation of lower brominated congeners. Recently, the concentrations of PBDEs in most environmental compartments are increasing markedly [2].

Due to the large surface area and high reaction activity, nanoscale zero-valent iron (nZVI) is becoming an increasingly popular choice for treatment of hazardous and toxic wastes [10], including effective debromination of PBDEs [11–13]. Some useful generalizations concerning reductive dehalogenation can be made from the studies on their corresponding anions. For example, Arulmozhiraja and Morita performed a study on the anionic dibenzofurans (PCDFs), in which the chlorine atoms at a specific substituted position were bent as the C–Cl bond stretches, and the reductive dechlorination could take place through the π*–σ* orbital mixing [14]. Furthermore, Zhao *et al*. investigated the possible dominant reductive dechlorination products of 1,2,3,7,8-PeCDD via a radical anion calculation, which were found to be in agreement with reported experimental results by the use of electron capture negative ion mass spectrometry (ECNI-MS) [15]. In both of the above cases, the significantly elongated carbon-halogen bonds at specific position were adopted as the probe to elucidate regioselectivity of reductive dehalogenation. Previous researchers have found that the reaction rates for the debromination of BDE congeners by zerovalent iron correlated well with its lowest unoccupied molecular energy (*E*_LUMO_) and electron affinity [13,16,17], and therefore, it is generally believed that this reaction probably occurs by an electron transfer mechanism. It could be inferred that the study on the anions can also provide valuable insight into the reductive dehalogenation of similar halogenated compounds in the presence of nZVI, due to the possible similar mechanism shared by ECNI-MS and nZVI. However, limited research has been done on the PBDEs radical anions. Hitherto, only Zhao *et al*. calculated four anionic BDE congeners mainly of the ortho-and para-bromination [17], and the elongated C–Br bonds were also found in the specific substituted position where the reductive debromination was observed experimentally [16].

PBDEs are chemically stable in general, however the reactivity of their congeners or in different substituted position may vary considerably [18,19]. Since experimental determination on debromination of all 209 PBDE congeners is an expensive job, generally low cost theoretical evaluation becomes a convenient assistant method based on the limited experimental data available [19,20]. Recently, debromination pathways and kinetics of PBDEs with nZVI were reported by Zhuang *et al*. for 2,3,4-tribromodiphenyl ether (BDE-21), which has one bromine at each of the ortho-, meta-, and para-positions on one side of diphenyl ether, and its daughter debromination products [13]. To better understand this reaction, in this paper we selected for study all seven BDE congeners involved in Reference [13]: 2-monobromodiphenyl ether (BDE-1), 3-monobromodiphenyl ether (BDE-2), 4-monobromodiphenyl ether (BDE-3), 2,3-dibromodiphenyl ether (BDE-5), 2,4-dibromodiphenyl ether (BDE-7), 3,4-dibromodiphenyl ether (BDE-12) and BDE-21. The geometries for these BDE congeners were investigated at the density functional theory (DFT) level and the same calculations were also performed for their anionic forms. Prediction of the dominant debromination products based on the elongated C–Br bond in the obtained equilibrium structures of anionic PBDEs were used to compare with the reported experimental results. In addition, frontier orbital energies and adiabatic electronic affinities of the selected BDE congeners were also investigated, and employed for correlation study with their reaction rate constant for the debromination by nZVI.

## 2. Results and Discussion

### 2.1. Use of Optimized Geometries of BDE Congeners for Prediction of Dominant Debromination Products by nZVI

**Figure 1:**
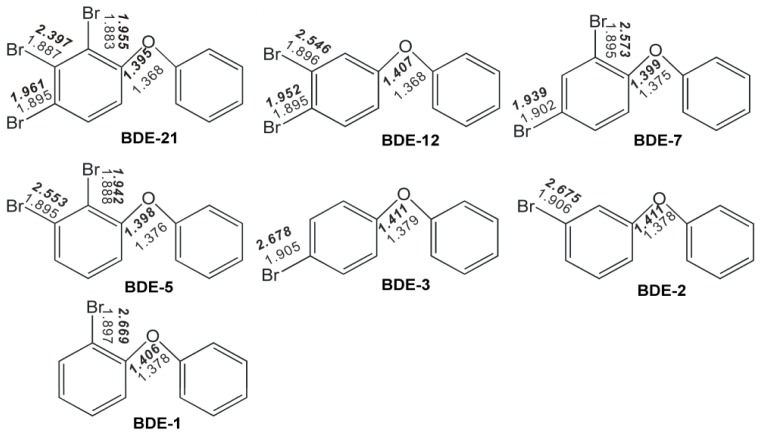
Structures and some important geometrical parameters of BDE neutrals and their anionic species (parameters in bold are shown for anionic PBDEs) from B3LYP/6-31+G(d) calculations (bond distances are given in angstroms).

**Figure 2:**
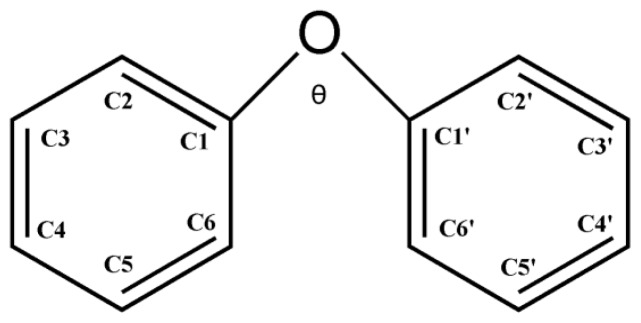
A schematic geometry of diphenyl ether and definitions of the atomic positions.

We present the geometries of selected BDE congeners obtained from B3LYP/6-31+G(d) in Figure 1 (Cartesian coordinates for the optimized structures from all our calculations are shown in Supplementary A). Presently, no structural data based on X-ray diffraction are available for the BDE congeners selected in this paper. Thus, we listed the previous reported theoretical calculated geometric parameters of BDE-7 and the corresponding results from the four methods in the present work in Table 1 for a comparison (the schematic geometry is shown in Figure 2), which shows that values of the geometric parameters obtained from these methods are fairly close. Previously, Zhao *et al*. reported that the molecular geometries of BDE neutral and its anionic species resulting from the two methods, B3LYP/6-31+G(d) and B3LYP/aug-cc-pVDZ, are virtually identical [17]. Thus, with the B3LYP hybrid functional, the basis set effects are not obvious on the geometrical structure of these selected BDE molecules.

**Figure 3:**
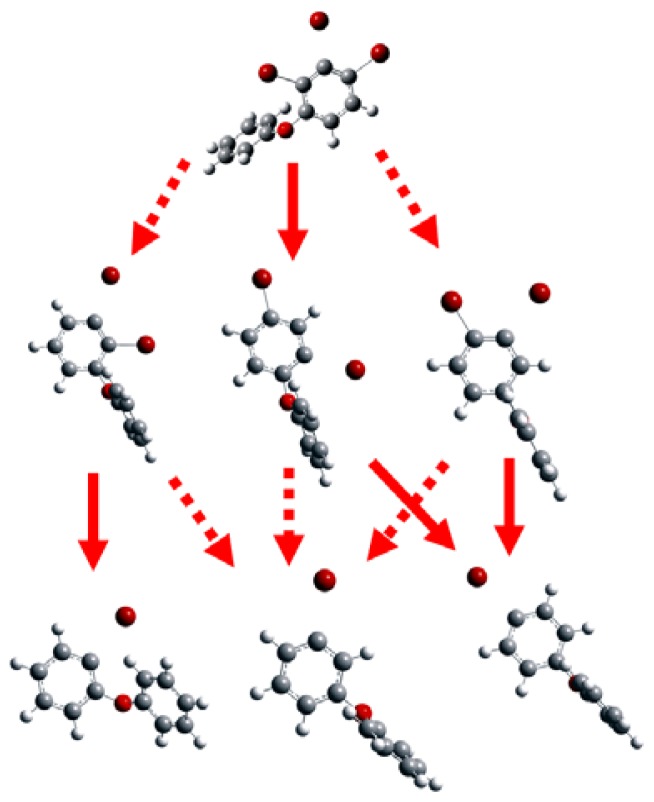
Visualized geometries (directly from GaussView 4.1) of the selected anionic BDE congeners optimized at the B3LYP/6-31+G(d) level and the proposed debromination pathway in this study (broader arrows indicate major pathways).

Because of the electron negative character of halogen substituents, polyhalogenated compounds could behave as electron acceptors or oxidants [21]. Anionic BDE congeners can be assumed as the molecules which have accepted the electrons from nZVI in the reductive environment. It was found that one of the C-Br bonds at the specific position of each anion of BDE congeners is approximately 0.6–0.9 Å longer than those of the corresponding neutral compounds (Figure 1). This means that the bond was significantly weakened by the added electron and is broken or cleaved within the context of covalent bonding (the visualized geometries directly from GaussView 4.1 (Semichem Inc., Shawnee Mission, KS, USA) are shown in Figure 3). Bending of this cleaved C–Br bonds out of the aromatic ring plane was also somehow observed in our calculations, implicating the π*–σ* orbital mixing [14,15]. Equilibrium geometry is the geometry at the energy minimum on the potential energy surface. Changing the geometry relative to the equilibrium structure must increase the energy. When the change occurs on the potential energy surface of the anion, the neutral-anion energy separation will decrease. This relationship can break down in pathological cases in which the nuclear configuration dramatically changes upon the addition of an electron [22]. The equilibrium nuclear configuration of the PBDE anion might correspond to some point along the pathway to dissociation of the neutral molecule. When an electron was added into the PBDE molecule, the susceptible debromination position could be inferred from the substituted position where the cleavage of C–Br bond occurred in our calculations. Based on this, the predicted dominant debromination products in this study (Figure 3) were proposed and compared with the main debromination pathway of PBDEs with nZVI reported by Zhuang *et al*. [13]. According to our proposed main debromination pathway of BDE-21 (Figure 3), the relative abundance of mono-BDE congeners should descend with the following order: BDE-3 > BDE-1 > BDE-2, and this trend was actually observed experimentally [13]. Most of our calculational results were consistent with the experimental debromination pathway except for BDE-7. The calculated debromination products indicated that meta-is the first primary position of the selected PBDE molecules for the cleavage of the C–Br bond to occur, clearly pointing to a meta-susceptibility which was observed experimentally by the previous researchers [13,16]. Ortho-is the relatively preferential position for debromination compared with the para-. This predicted para-positional resistance to reductive debromination is also in agreement with the earlier reports by Shih *et al*. [11] and by Keum and Li [16]. In addition, such a positional preference is different from the debromination of PBDEs by nZVI/Pd, which was assumed to be through a mechanism concerning hydrogen atom transfer, thus its positional preference is probably dominated by steric hindrance [23].

As indicated above, a priori prediction based on the calculated equilibrium structure satisfactorily conform to the experimental results, which provides a convenient method for the prediction of the major debromination pathway of PBDEs in some specific systems which concern the electron transfer mechanism. This outcome can also indicate that this kind of the PBDE debromination may be dominated by a thermodynamic process as reported by the previous researchers [16], meaning that this reaction favored to more stable products, *i.e.*, low heat of formation (*H*_f_) [19] (the thermodynamic properties of these BDE congeners from our calculations are listed in Table S1). However, other factors, such as steric effect [13] and the net charge at different brominated positions [12] might also play a role in this debromination process.

Calculation of the divalent anion of BDE-21 was also performed at the B3LYP/6-31G(d) level in the present research, and the results indicated that no double debromination takes place, proving the theory of a stepwise debromination in general [16]. Nevertheless, the double debromination was observed in the calculation of the triple-valent anion of BDE-21 (not properly converged), indicating that a multiple debromination pathway is possible if electrons are highly oversupplied (*i.e.*, use of excessive amount of ZVI). Interestingly, no cleavage of C–O linkage was found in any of the above calculations including those for divalent and triple valent ions (actually indicating strong reductive conditions). Implication of this finding is three-fold: (1) Persistency of PBDEs (probably also including dibenzodioxin and dibenzofuran types of compounds) comes partly from this highly stable ether linkage; (2) PBDEs have become so widespread in the environment partially because their degradation may be mostly due to a slow stepwise debromination, not to the cleavage of the ether linkage; (3) The reductive debromination (e.g., by ZVI) is of its limitation since it may not damage the framework of BDE congeners readily.

### 2.2. Frontier Orbital Energies, Adiabatic Electron Affinity and Their Relevance to Kinetics of Debromination Reactions by nZVI

**Figure 4:**
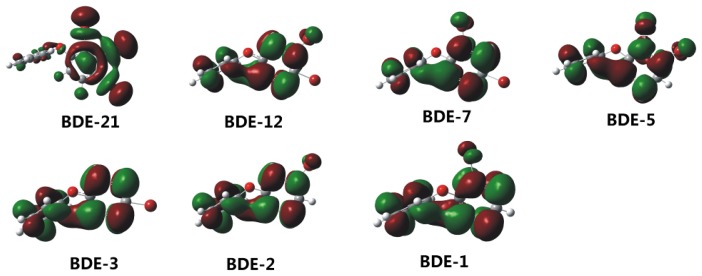
Surface images of lowest unoccupied molecular orbital (LUMO) of the neutral BDE congeners (taking the results obtained at the B3LYP/6-311G(d,p) level as an example).

According to frontier orbital theory [24], frontier orbital characteristics of reacting molecules, namely the highest occupied molecular orbital (HOMO) and the lowest unoccupied molecular orbital (LUMO), determine the kinetics of a chemical reaction. Susceptibility of the position toward debromination reaction could be viewed as favoring nucleophilic substitution; the combined effects of the ether bond and the bromines on the electron density of a phenyl ring may make the specific-bromine more vulnerable to electron attack relatively [19]. Spatial distributions of the LUMO obtained from the four methods are compatible for the BDE congeners in this study. The LUMO is almost completely located on the brominated phenyl group (Figure 4, take the surface image from 6-311G(d,p) basis set as an example), implicating reasonably that reductive reactions should occur to this phenyl group [19] and a sigmatropic bromine shift within the phenyl group be possible. Furthermore, as shown in Table 2, *E*_LUMO_ of the neutral BDE congeners tends to decrease with an increasing number of the bromine substituents, implicating that an increase in bromine number would lead to the increasing reductive reactivity or electrophilicity of PBDEs. Satisfactory linear relationships were observed between the logarithm of the reductive debromination rate constants of PBDEs with nZVI [13] and *E*_LUMO_, the energy of HOMO (*E*_HOMO_) and the HOMO-LUMO energy gap (see the calculated values in Tables S2–S5 and the Pearson correlation analysis in Table S6). The linear relationship based on *E*_LUMO_ is significantly better than that based on the other parameters calculated. The *E*_LUMO_ values calculated using B3LYP/6-31G(d) and B3LYP/6-31G(d,p) have the best statistical quality in the linear regression analysis both with the squared regression coefficient value of 0.961 (Table 2). These results are in agreement with the previous studies using E_LUMO_ values from AM1 semiempirical method [13,16] and with the correlation analysis of rate constants for the dehalogenation of other compounds with zero valent iron [25].

**Figure 5:**
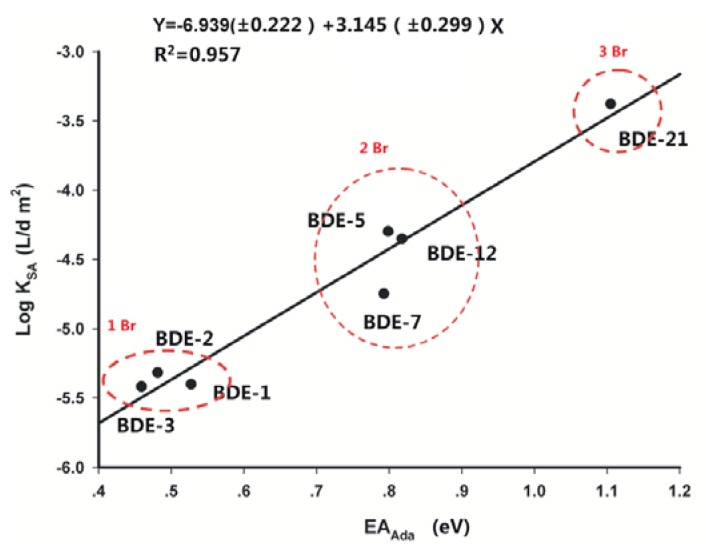
Correlation between debromination rate constants and *EA*_Ada_ values from B3LYP/6-311G(d,p).

The energy difference between an uncharged species and its negative ion, referred to as an electron affinity, is an important property of molecules. The theoretical *EA*_Ada_ represents the difference between the total energies of the neutral and anion at their respective equilibrium nuclear configurations [22]. Table 2 lists the theoretically evaluated *EA*_Ada_ values from the B3LYP calculation with the four basis sets, and but does not include the corresponding experimental results since, to our knowledge, no experimental *EA*_Ada_ values for these selected PBDEs are available. The calculated *EA*_Ada_ values of PBDEs may be slightly lower than that calculated with a relatively large basis set. For example, the *EA*_Ada_ value calculated with B3LYP\6-31+G(d) was on average 0.2 eV lower than that from B3LYP\Aug-ccpVDZ [17]. A positive linear relationship was also found between the *EA*_Ada_ and the rate constants of the reductive debromination [13], as shown in Table 2. The calculated *EA*_Ada_ values of the BDE congeners with different number of the brominated substituents seemed to differ notably. With each bromine atom added to the BDE congener, the calculated *EA*_Ada_ values increased by approximately 0.3 eV and the observed rate constant decreased by about 10 times correspondingly. *EA*_Ada_ values calculated using B3LYP/6-311G(d,p) have the best statistical quality in the correlation analysis with the observed rate constants (the obtained linear relationship is shown in Figure 5).

Electron affinity is related to the *E*_LUMO_ and could also use as an indicator of the susceptibility of the molecule toward reductive or nucleophilic attack. The calculated *EA*_Ada_ values are positive or near zero, suggesting that these PBDEs might act as electron acceptors in charge-transfer interactions. Both the significant linear relationship obtained above and the studies on the BDE anions clearly showed the evidence of an electron transfer mechanism associated with the debromination of PBDEs by nZVI, however such relationship seemed nonexistent in the cases of the dechlorination of PCBs [26,27] and PCDDs [28], and debromination of PBDEs with the palladized material in which the precursor complex formation rather than electron transfer is the rate-controlling step.

## 3. Experimental Section

All calculations were carried out with the Gaussian03 program suite [29]. GaussView 4.1 was used as the molecular modeling system for constructing and visualizing the results of the calculations with Gaussian03. The molecular geometries of target BDE neutrals and their corresponding anions were optimized using B3LYP hybrid functional of DFT [30,31] in conjunction with four basis sets with polarization functions, including 6-31G(d), 6-31+G(d), 6-31G(d,p) and 6-311G(d,p). Due to the use of the DFT method, spin contamination for open-shell anions was small [32]. The maximum expectation value of the *S*^2^ operator for doublets in this study is less than 0.76. The optimized geometries were confirmed by the harmonic vibrational frequencies to ensure that all geometries obtained are true energy minima not saddle points. These obtained vibrational frequencies were used to calculate the zero-point energies (ZPE), which were needed for adiabatic electron affinity (*EA*_Ada_) calculations. *EA*_Ada_ were obtained from the following relationship:

$$EA_{\text{Ada}}=E_{\text{neutral}}\;(\text{optimized neutral})-E_{\text{anion}}\;(\text{optimized anion})$$

Statistical analyses were undertaken with the use of PASW 18 for Windows (SPSS Inc., Chicago, IL, USA).

## 4. Conclusions

In the present work, using the cleaved C–Br bond of the anionic BDE congeners from the DFT calculations as the probe of the susceptible debromination position gave a satisfactory predictive ability for the dominant debromination products of PBDEs by nZVI. The debromination preference was theoretically evaluated as meta-Br > ortho-Br > para-Br. In addition, significant correlations were found between the reductive debromination rate constant of PBDEs with nZVI, and their frontier orbital energies (especially *E*_LUMO_) and adiabatic electron affinities. These results clearly showed the evidence of an electron transfer mechanism associated with this reductive debromination reaction, based on which a deepened understanding on the environmental behaviors of such halogenated aromatic compounds can be obtained, since the photodebromination of these compounds may also involve the same mechanism [33].

## Supplementary Information



## Figures and Tables

**Table 1:** Geometrical parameters of neutral species and anions of 2,4-dibromodiphenyl ether (BDE-7) calculated with different basis sets with B3LYP hybrid functional (bond distances are given in angstroms, angles are given in degrees).

	6-31G(d)		6-31+G(d)		6-31G(d,p)		6-311G(d,p)		6-31+G(d) a	aug-cc-pVDZ a
						
	neutral	anion	neutral	anion	neutral	anion	neutral	anion	neutral	neutral
C1–O	1.368	1.400	1.375	1.399	1.368	1.399	1.365	1.398	1.375	1.370
C1′–O	1.388	1.374	1.388	1.370	1.388	1.373	1.39	1.369	1.388	1.391
C1–Br	1.902	2.703	1.895	2.573	1.902	2.695	1.908	2.698	1.895	1.909
C3–Br	1.910	1.946	1.902	1.939	1.910	1.946	1.916	1.952	1.902	1.971
C1–O–C1′	120.6	123.0	120.4	120.7	120.6	123.5	120.1	123.1	120.4	120.3

a Cited from reference [17].

**Table 2:** *E*_LUMO_ (hartree) and *E*A_Ada_ (eV) of selected BDE congeners obtained with the B3LYP hybrid functional.

IUPAC no.	6-31G(d)		6-31+G(d)		6-31G(d,p)		6-311G(d,p)		Log K_SA_ a
			
	*E*_LUMO_	*EA*_Ada_	*E*_LUMO_	*EA*_Ada_	*E*_LUMO_	*EA*_Ada_	*E*_LUMO_	*EA*_Ada_	
BDE21	−0.0404	0.6481	−0.0527	0.9558	−0.0404	0.6509	−0.0544	1.1048	−3.378
BDE12	−0.0279	0.3131	−0.0439	0.6708	−0.0284	0.3142	−0.0400	0.8174	−4.351
BDE7	−0.0286	0.3707	−0.0471	0.7106	−0.0291	0.3743	−0.0402	0.7926	−4.747
BDE5	−0.0299	0.3219	−0.0437	0.6817	−0.0303	0.3231	−0.0375	0.7983	−4.298
BDE3	−0.0196	−0.0394	−0.0363	0.3549	−0.0204	−0.0398	−0.0325	0.4589	−5.417
BDE2	−0.0188	−0.0195	−0.0358	0.3701	−0.0196	−0.0194	−0.0315	0.4812	−5.317
BDE1	−0.0198	0.0969	−0.0357	0.4491	−0.0204	0.1029	−0.0300	0.5272	−5.401
R^2^^b^	0.961	0.896	0.863	0.915	0.961	0.892	0.896	0.957	-

a Log K_SA_ is the logarithm of the debromination constant of selected PBDEs normalized by the surface area of nZVI, taken from reference [13];

b *R*^2^ is the squared linear regression coefficient (*n* = 7).
