# Supplementary material for: A Theoretical Study on Reductive Debromination of Polybrominated Diphenyl Ethers
Source: Int J Mol Sci. 2012 Jul 24;13(7):9332–42. doi: 10.3390/ijms13079332 (PMC3430299; doi:10.3390/ijms13079332)
Supplement: Supplementary file 1 [file ijms-13-09332-s001.pdf]

Supplementary Information

A Theoretical Study on Reductive Debromination of Polybrominated Diphenyl Ethers

Ji-Wei Hu [1,]*, Yuan Zhuang [2], Jin Luo [1], Xiong-Hui Wei [3] and Xian-Fei Huang [1]

Table S1. Thermodynamic data (hartrees) of PBDEs from our calculations (298.150 K, 1. 00000 atm).

	**Enthalpy**				**Gibbs free energy**			
	6-311G(d,p)	6-31+G(d)	6-31G(d,p)	6-31G(d)	6-311G(d,p)	6-31+G(d)	6-31G(d,p)	6-31G(d)
BDE-21	−8259.086562	−8251.736078	−8251.655962	−8251.644565	−8259.149517	−8251.795865	−8251.718253	−8251.706708
BDE-12	−5685.546311	−5680.605631	−5680.551702	−5680.538672	−5685.604327	−5680.662728	−5680.609247	−5680.596192
BDE-7	−5685.547892	−5680.607363	−5680.551598	−5680.538532	−5685.606217	−5680.663558	−5680.609181	−5680.596103
BDE-5	−5685.543345	−5680.60505	−5680.549172	−5680.536119	−5685.601776	−5680.661627	−5680.606043	−5680.593017
BDE-3	−3112.001396	−3109.47206	−3109.443713	−3109.429089	−3112.058179	−3109.526053	−3109.496814	−3109.482163
BDE-2	−3112.001668	−3109.472846	−3109.443961	−3109.429327	−3112.054704	−3109.525658	−3109.496709	−3109.482064
BDE-1	−3111.998593	−3109.471522	−3109.441435	−3109.426781	−3112.05202	−3109.523349	−3109.493982	−3109.479368
BDE-21 anion	−8259.126329	−8251.770113	−8251.678918	−8251.667408	−8259.19229	−8251.833864	−8251.744878	−8251.733431
BDE-12 anion	−5685.575379	−5680.629398	−5680.562203	−5680.549123	−5685.638644	−5680.690082	−5680.625026	−5680.612042
BDE-7 anion	−5685.57612	−5680.632546	−5685.57612	−5680.552146	−5685.638132	−5680.692491	−5685.638132	−5680.610463
BDE-5 anion	−5685.571815	−5680.629238	−5680.560039	−5680.54694	−5685.633391	−5680.689333	−5680.621758	−5680.60866
BDE-3 anion	-3112.017345	-3109.484227	−3109.441197	−3109.426588	−3112.075228	−3109.540723	−3109.499673	−3109.485089
BDE-2 anion	−3112.018317	−3109.485511	−3109.442152	−3109.427501	−3112.077106	−3109.54267	−3109.501071	−3109.486678
BDE-1 anion	−3112.017042	−3109.487249	−3109.444273	−3109.429419	−3112.07389	−3109.542545	−3109.501118	−3109.48605

Table S2. EA_{Ada} (eV), E_{LUMO} (hartree), E_{HOMO} (hartree) and HOMO-LUMO energy gap (hartree) of selected BDE congeners calculated at B3LYP/6-31G(d) level.

	EA_{Ada}	E_{LUMO}	E_{HOMO}	**HOMO-LUMO energy gap**
BDE21	0.6481	−0.04043	−0.23027	0.18984
BDE12	0.3131	−0.02786	−0.22691	0.19905
BDE7	0.3707	−0.02856	−0.22628	0.19772
BDE5	0.3219	−0.02986	−0.22785	0.19799
BDE3	−0.0394	−0.01957	−0.22032	0.20075
BDE2	−0.0195	−0.01879	−0.22552	0.20673
BDE1	0.0969	−0.01976	−0.22347	0.20371

Table S3. EA_{Ada} (eV), E_{LUMO} (hartree), E_{HOMO} (hartree) and HOMO-LUMO energy gap (hartree) of selected BDE congeners calculated at B3LYP/6-31+G(d) level.

6-31+G(d)	EA_{Ada}	E_{LUMO}	E_{HOMO}	HOMO-LUMO energy gap
BDE21	0.9558	−0.05265	−0.24088	0.18823
BDE12	0.6708	−0.04394	−0.23686	0.19292
BDE7	0.7106	−0.04713	−0.23961	0.19248
BDE5	0.6817	−0.04368	−0.23867	0.19499
BDE3	0.3549	−0.0363	−0.23042	0.19412
BDE2	0.3701	−0.03584	−0.23629	0.20045
BDE1	0.4491	−0.03572	−0.23452	0.1988

Table S4. EA_{Ada} (eV), E_{LUMO} (hartree), E_{HOMO} (hartree) and HOMO-LUMO energy gap (hartree) of selected BDE congeners calculated at B3LYP/6-31G(d,p) level.

6-31G(d,p)	EA_{Ada}	E_{LUMO}	E_{HOMO}	HOMO-LUMO energy gap
BDE21	0.65092	−0.04041	−0.2304	0.18999
BDE12	0.3142	−0.02847	−0.22703	0.19856
BDE7	0.3743	−0.02917	−0.2264	0.19723
BDE5	0.3231	−0.03028	−0.22819	0.19791
BDE3	−0.0398	−0.0204	−0.22049	0.20009
BDE2	−0.0194	−0.01957	−0.22563	0.20606
BDE1	0.1029	−0.02041	−0.22382	0.20341

Table S5. EA_{Ada} (eV), E_{LUMO} (hartree), E_{HOMO} (hartree) and HOMO-LUMO energy gap (hartree) of selected BDE congeners calculated at B3LYP/6-311G(d,p) level.

6-311G(d,p)	EA_{Ada}	E_{LUMO}	E_{HOMO}	HOMO-LUMO energy gap
BDE21	1.1048	−0.05436	−0.24049	0.18613
BDE12	0.8174	−0.03997	−0.23657	0.1966
BDE7	0.7926	−0.0402	−0.23636	0.19616
BDE5	0.7983	−0.03747	−0.24192	0.20445
BDE3	0.4589	−0.03245	−0.22986	0.19741
BDE2	0.4812	−0.03154	−0.23538	0.20384
BDE1	0.5272	−0.03001	−0.23269	0.20268

Table S6. Correlation matrix of the linear regression between the molecular parameters and the debromination rate constant.

	(1)	(2)	(3)	(4)	(5)	(6)	(7)	(8)	(9)	(10)	(11)	(12)	(13)	(14)	(15)	(16)	(17)
(1)	1																
(2)	−0.979	1															
(3)	−0.866	0.854	1														
(4)	−0.912	0.950	0.649	1													
(5)	0.999	−0.984	−0.872	−0.915	1												
(6)	−0.975	0.971	0.825	0.926	−0.981	1											
(7)	−0.856	0.806	0.949	0.610	−0.856	0.835	1										
(8)	−0.823	0.860	0.503	0.956	−0.831	0.880	0.474	1									
(9)	1.000	−0.977	−0.865	−0.910	0.998	−0.974	−0.856	−0.821	1								
(10)	−0.979	1.000	0.853	0.951	−0.984	0.974	0.806	0.863	−0.977	1							
(11)	−0.865	0.852	0.999	0.647	−0.871	0.819	0.947	0.493	−0.864	0.851	1						
(12)	−0.905	0.944	0.635	0.999	−0.909	0.926	0.598	0.966	−0.903	0.945	0.631	1					
(13)	0.991	−0.988	−0.883	−0.916	0.995	−0.975	−0.838	−0.838	0.990	−0.989	−0.880	−0.910	1				
(14)	−0.936	0.968	0.793	0.940	−0.939	0.954	0.737	0.890	−0.934	0.968	0.784	0.939	−0.953	1			
(15)	−0.774	0.788	0.932	0.593	−0.788	0.734	0.881	0.416	−0.772	0.787	0.939	0.575	−0.795	0.658	1		
(16)	−0.711	0.744	0.422	0.834	−0.706	0.760	0.382	0.886	−0.710	0.745	0.406	0.845	−0.720	0.870	0.202	1	
(17)	0.947	−0.980	−0.887	−0.901	0.956	−0.929	−0.786	−0.809	0.944	−0.980	−0.885	−0.894	0.978	−0.946	−0.826	−0.691	1

In Table S6, the values in the table are the regression coefficients in Pearson correlation analysis (*R*, *n* = 7). (1) represents the EA_{Ada} calculated at B3LYP/6-31G(d) level; (2) represents the E_{LUMO} calculated at B3LYP/6-31G(d) level; (3) represents the E_{HOMO} calculated at B3LYP/6-31G(d) level; (4) represents the HOMO-LUMO energy gap calculated at B3LYP/6-31G(d) level; (5) represents the EA_{Ada} calculated at B3LYP/6-31+G(d) level; (6) represents the E_{LUMO} calculated at B3LYP/6-31+G(d) level; (7) represents the E_{HOMO} calculated at B3LYP/6-31+G(d) level; (8) represents the HOMO-LUMO energy gap calculated at B3LYP/6-31+G(d) level; (9) represents the EA_{Ada} calculated at B3LYP/6-31G(d,p) level; (10) represents the E_{LUMO} calculated at B3LYP/6-31G(d,p) level; (11) represents the E_{HOMO} calculated at B3LYP/6-31G(d,p) level; (12) represents the HOMO-LUMO energy gap calculated at B3LYP/6-31G(d,p) level; (13) represents the EA_{Ada} calculated at B3LYP/6-311G(d,p) level; (14) represents the E_{LUMO} calculated at B3LYP/6-311G(d,p) level; (15) represents the E_{HOMO} calculated at B3LYP/6-311G(d,p) level; (16) represents the HOMO-LUMO energy gap calculated at B3LYP/6-311G(d,p) level; (17) represents the logarithm of observed debromination rate constant obtained from Zhuang *et al*. [1].

Reference

1. Zhuang, Y.; Ahn, S.; Luthy, R.G. Debromination of polybrominated diphenyl ethers by nanoscale zerovalent iron: pathways, kinetics, and reactivity. *Environ. Sci. Technol.* **2010**, *44*, 8236–8242.

Molecular geometries

The Cartesian coordinates of optimized PBDE geometries are shown as follows:

neutral BDE-21, B3LYP/6-311G(d,p)

C	0.10758700	0.72652300	−0.00012400
C	1.35588200	0.08807300	−0.00004900
C	1.40926800	−1.31340600	−0.00017600
C	0.23325800	−2.05381800	−0.00039500
C	−1.00217900	−1.42257600	−0.00048000
C	−1.07594100	−0.03156500	−0.00033300
O	−2.25118400	0.66045800	−0.00047800
C	−3.46293100	−0.02605300	−0.00014100
C	−4.07576500	−0.32407300	−1.21269500
C	−5.32529200	−0.94052100	−1.20629000
C	−5.94941500	−1.25183100	0.00050700
C	−5.32426200	−0.94132100	1.20699200
C	−4.07474900	−0.32487000	1.21275000
Br	−0.06059900	2.62187600	0.00003200
Br	2.95800000	1.11248700	0.00021800
Br	3.05816400	−2.27858600	−0.00007700
H	0.28551800	−3.13421800	−0.00051200
H	−1.91038000	−2.00954700	−0.00067700
H	−3.57953000	−0.06215800	−2.13929900
H	−5.81242400	−1.17221100	−2.14640100
H	−6.92288400	−1.72793600	0.00076900
H	−5.81061200	−1.17363400	2.14735400
H	−3.57774600	−0.06355000	2.13911100

anionic BDE-21, B3LYP/6-311G(d,p)

C	0.10607500	0.20675600	−0.63013800
C	−1.21798100	0.00327700	−0.32903300
C	−1.68075800	−1.28491600	−0.26045000
C	−0.85540700	−2.39018300	−0.46210500
C	0.49146800	−2.17176600	−0.73492100
C	0.97741500	−0.86888000	−0.82730900
O	2.31213600	−0.69053200	−1.18977200
C	3.27694500	−0.65147400	−0.22007500
C	3.01057600	−0.65542200	1.15078300
C	4.07234600	−0.61784100	2.05245900
C	5.39174700	−0.57658300	1.60847800
C	5.64594700	−0.56739800	0.23622600
C	4.59852700	−0.60432000	−0.67579800
Br	0.83916200	2.00917800	−0.86196500
Br	−2.54157800	1.84989400	0.79113400
Br	−3.57808700	−1.68900400	0.07964900
H	−1.25145500	−3.39804300	−0.41177300
H	1.17426800	−2.99885400	−0.89170300
H	1.98754800	−0.67423700	1.50217100
H	3.85692200	−0.61440300	3.11571900
H	6.21002000	−0.54616900	2.31918900
H	6.66758800	−0.53028000	−0.12792700
H	4.77422700	−0.59365400	−1.74484600

neutral, BDE-21 B3LYP/6-31G(d)

C	−0.08889700	0.70012000	−0.10416600
C	−1.35133400	0.09254200	−0.04103400
C	−1.44823200	−1.30324800	−0.12759100
C	−0.29868000	−2.07422900	−0.28245500
C	0.95233200	−1.47303200	−0.35157300
C	1.06690900	−0.08460700	−0.25787700
O	2.25780900	0.57753500	−0.36093300
C	3.45372500	−0.08492800	−0.10756300
C	3.72171700	−0.62971500	1.14981200
C	4.96729200	−1.21268400	1.38327500
C	5.93542600	−1.24135500	0.37615000
C	5.65473400	−0.68291600	−0.87212200
C	4.40920300	−0.10376500	−1.12156000
Br	0.11733100	2.58091600	0.01020600
Br	−2.90896400	1.14930100	0.16535700
Br	−3.12238300	−2.20242600	−0.03982600
H	−0.38844800	−3.15194700	−0.35543600
H	1.84212600	−2.07804900	−0.48102700
H	2.96675600	−0.58743800	1.92871900
H	5.18353700	−1.63687700	2.35986400
H	6.90490900	−1.69275600	0.56587700
H	6.40454500	−0.69795100	−1.65814200
H	4.16948800	0.33893700	−2.08301000

anionic BDE-21, B3LYP/6-31G(d)

C	0.11506700	0.09899800	−0.59975600
C	−1.21764800	−0.01552300	−0.29834900
C	−1.75715400	−1.27008200	−0.18307500
C	−1.00087900	−2.43142400	−0.34707600
C	0.35939800	−2.30299300	−0.63010100
C	0.92337200	−1.03181400	−0.76432100
O	2.26283100	−0.95630300	−1.14889000
C	3.23435700	−0.74309300	−0.20634500
C	4.55465500	−0.82210800	−0.66756600
C	5.61277700	−0.63547100	0.21805500
C	5.36909100	−0.36993600	1.56852600

C	4.04951200	−0.28837900	2.01668700
C	2.97771600	−0.47205100	1.14201900
Br	0.95806600	1.85228000	−0.84848400
Br	−2.43451300	1.91556800	0.59409200
Br	−3.67527400	−1.52902100	0.19068700
H	−1.45948900	−3.41272400	−0.25854700
H	0.99391000	−3.17566800	−0.75645700
H	4.72374200	−1.02893200	−1.72005600
H	6.63429900	−0.69960600	−0.15056900
H	6.19546500	−0.22356500	2.25925100
H	3.84265900	−0.07264000	3.06227900
H	1.95540900	−0.39211900	1.49268800

neutral BDE-21, B3LYP/6-31+G(d)

C	0.10874600	0.72672800	−0.00018200
C	1.35486300	0.08358500	−0.00001000
C	1.40967800	−1.31768200	−0.00023400
C	0.23162100	−2.06080600	−0.00054800
C	−1.00523600	−1.42608800	−0.00083500
C	−1.07280800	−0.03198100	−0.00055700
O	−2.24702200	0.66920500	−0.00084400
C	−3.45901300	−0.01779500	−0.00020200
C	−4.07365300	−0.31219200	−1.21690700
C	−5.32769400	−0.92918400	−1.20983700
C	−5.95501700	−1.23939700	0.00090300
C	−5.32615000	−0.93014900	1.21107900
C	−4.07206300	−0.31322000	1.21705500
Br	−0.04208200	2.60408300	−0.00010100
Br	2.94368000	1.10112300	0.00050300
Br	3.05383600	−2.26074800	−0.00018600
H	0.28312200	−3.14392100	−0.00075600
H	−1.91637700	−2.01308500	−0.00117800
H	−3.57510700	−0.05229300	−2.14605000
H	−5.81685100	−1.16028100	−2.15241700
H	−6.93196400	−1.71492800	0.00133500
H	−5.81410300	−1.16198300	2.15410300
H	−3.57237600	−0.05410500	2.14580300

anionic BDE-21, B3LYP/6-31+G(d)

C	0.12107600	0.19214900	−0.48548400
C	−1.19657000	0.00766100	−0.16185400
C	−1.72032300	−1.25690600	−0.21981100
C	−0.95780600	−2.36611900	−0.59353100
C	0.38708600	−2.17230100	−0.90948700
C	0.92903100	−0.88724400	−0.85764800
O	2.25441700	−0.71659600	−1.25763100
C	3.24980200	−0.67008300	−0.31687000
C	4.52966700	−0.36632000	−0.79993900
C	5.61515200	−0.34593600	0.07362100
C	5.43746500	−0.61506600	1.43632400
C	4.15542000	−0.90889400	1.90787100
C	3.05654800	−0.93507900	1.04410500
Br	0.95461200	1.96067500	−0.45974800
Br	−2.58208500	1.86676900	0.44596500
Br	−3.59817800	−1.62278400	0.21001400
H	−1.39859700	−3.35898600	−0.63719400
H	1.02200900	−3.00249500	−1.20787100
H	4.64843500	−0.15041700	−1.85773300
H	6.60485500	−0.10863300	−0.31113800
H	6.28416100	−0.59351000	2.11792400
H	3.99825900	−1.11351300	2.96464500
H	2.06404500	−1.15791400	1.42055500

neutral BDE-12, B3LYP/6-311G(d,p)

C	−0.07158600	1.21874900	−0.09899300
C	−1.32233700	0.61294100	−0.03830400
C	−1.43522400	−0.77814000	−0.12630200
C	−0.28026700	−1.54224000	−0.28274900
C	0.97354900	−0.94668800	−0.35032400
C	1.07495100	0.44121500	−0.25458600
O	2.25625500	1.13237800	−0.35566500
C	3.46451300	0.49563500	−0.10415200
C	3.74026300	−0.05755800	1.14493800
C	4.99298000	−0.61953600	1.37608000
C	5.96243200	−0.62143300	0.37373400
C	5.67569100	−0.05531400	−0.86645500
C	4.42288300	0.50313600	−1.11212300
Br	−3.11348900	−1.68449000	−0.04290200
Br	−2.84350600	1.74622900	0.17536700
H	0.02703500	2.29339800	−0.03076100
H	−0.36676400	−2.61831700	−0.35821900
H	1.85681400	−1.55729000	−0.48007600
H	2.98523500	−0.03885600	1.92166000
H	5.21379100	−1.04894800	2.34662100
H	6.93705800	−1.05629300	0.56137000
H	6.42615100	−0.04898100	−1.64857000
H	4.17839300	0.95078600	−2.06756200

anionic BDE-12, B3LYP/6-311G(d,p)

C	0.23873000	0.45992500	−0.69944900
C	−1.06971100	0.19960700	−0.31064500
C	−1.47120700	−1.09556600	−0.13824600
C	−0.61441100	−2.17863300	−0.34295800

C	0.70065900	−1.92534900	−0.72862100
C	1.11280500	−0.60522300	−0.90073000
O	2.42354400	−0.36800300	−1.35056200
C	3.41529000	−0.12965600	−0.44348400
C	3.22546200	−0.10170000	0.94131500
C	4.31390200	0.14503200	1.77610300
C	5.58676500	0.36334600	1.25478200
C	5.76587900	0.33585000	−0.12920200
C	4.69168300	0.09197200	−0.97535700
Br	−3.31281700	−1.52849300	0.41329700
Br	−2.40602300	2.47126000	−0.02939500
H	0.57786200	1.48051300	−0.85131700
H	−0.96046400	−3.19786800	−0.20692400
H	1.39946000	−2.73507800	−0.90443900
H	2.23764300	−0.26055700	1.35253100
H	4.15591800	0.16934600	2.84925500
H	6.42550200	0.55623000	1.91420100
H	6.74960300	0.50753100	−0.55421600
H	4.81042400	0.06946100	−2.05221200

neutral BDE-12, B3LYP/6-31G(d)

C	−0.05740100	1.20819000	−0.13112400
C	−1.31381200	0.61164400	−0.05058500
C	−1.44328300	−0.77735900	−0.15538400
C	−0.30069700	−1.55516400	−0.34894000
C	0.95934700	−0.97024900	−0.43745500
C	1.07801200	0.41827500	−0.32351400
O	2.26697000	1.09456300	−0.45224000
C	3.46661700	0.47138800	−0.13619400
C	3.67279100	−0.12816800	1.10870500
C	4.92397200	−0.67098600	1.40174300
C	5.96091600	−0.60734100	0.46774700
C	5.74242200	0.00453400	−0.76798200
C	4.49271300	0.54348400	−1.07735100
Br	−3.13088000	−1.64672400	−0.04168800
Br	−2.82459300	1.73619600	0.21384200
H	0.04999500	2.28319100	−0.04800800
H	−0.40442900	−2.63100300	−0.43751600
H	1.83674900	−1.58651500	−0.59630200
H	2.86569800	−0.16115900	1.83382000
H	5.08938100	−1.13734100	2.36911200
H	6.93371100	−1.02877100	0.70382300
H	6.54451700	0.06081000	−1.49869100
H	4.30061500	1.02330500	−2.03173500

anionic BDE-12, B3LYP/6-31G(d)

C	0.24431700	0.51849800	−0.68787800
C	−1.06347700	0.23914000	−0.30261600
C	−1.44747900	−1.06462100	−0.14418900
C	−0.57981100	−2.13939900	−0.35508200
C	0.73514400	−1.86716700	−0.73877300
C	1.13273800	−0.53723500	−0.89913100
O	2.43985500	−0.28217400	−1.35305600
C	3.44102300	−0.08037500	−0.44604800
C	3.25794000	−0.07281600	0.94268400
C	4.35685800	0.13923400	1.77714700
C	5.63386200	0.34272700	1.25206000
C	5.80589300	0.33561000	−0.13556700
C	4.72109500	0.12612200	−0.98207100
Br	−3.28703400	−1.51220200	0.40275100
Br	−2.50247400	2.36537500	−0.01354000
H	0.57418200	1.54680400	−0.82954500
H	−0.91768200	−3.16512800	−0.22619200
H	1.44595700	−2.66830500	−0.92112600
H	2.26709400	−0.22235600	1.35573500
H	4.20452100	0.14703100	2.85404700
H	6.48169400	0.50748500	1.91189300
H	6.79319300	0.49459000	−0.56402400
H	4.83635900	0.11791400	−2.06192500

neutral BDE-12, B3LYP/6-31+G(d)

C	−0.05216300	1.19842400	−0.16336800
C	−1.31264200	0.60973400	−0.06479300
C	−1.45430100	−0.77852100	−0.16652200
C	−0.31835600	−1.56510700	−0.37319400
C	0.94572200	−0.99045300	−0.47688900
C	1.07277800	0.39731500	−0.36262800
O	2.26759000	1.06115600	−0.52048200
C	3.46201700	0.44885800	−0.16405000
C	3.64490400	−0.11116600	1.10342500
C	4.89515700	−0.63472600	1.44031100
C	5.95437400	−0.58749700	0.52791800
C	5.75843000	−0.01040100	−0.72997200
C	4.50880900	0.50547100	−1.08420900
Br	−3.13776800	−1.63697600	−0.02429800
Br	−2.80309700	1.74550800	0.22155800
H	0.06617400	2.27337200	−0.08693700
H	−0.42750200	−2.64112400	−0.46017300
H	1.81675400	−1.61361200	−0.64723400
H	2.82332000	−0.12962400	1.81333700
H	5.04269500	−1.07079600	2.42497000
H	6.92595700	−0.99151800	0.79847900
H	6.57748600	0.03544300	−1.44293100

H	4.33631500	0.95842100	−2.05591100

anionic BDE-12, B3LYP/6-31+G(d)

C	0.23625400	0.50734700	−0.68375500
C	−1.07846100	0.24802100	−0.30450000
C	−1.48013300	−1.05059300	−0.12865500
C	−0.61473700	−2.13340400	−0.31927300
C	0.70617800	−1.88387100	−0.69900700
C	1.11491600	−0.55958600	−0.87116700
O	2.42108800	−0.31891200	−1.33673400
C	3.43362100	−0.10839800	−0.44322100
C	3.26713100	−0.10016700	0.94835600
C	4.37630300	0.12539100	1.76939900
C	5.64608200	0.33978700	1.22703300
C	5.79994800	0.33113100	−0.16494900
C	4.70461600	0.11045600	−0.99759500
Br	−3.31151500	−1.48557300	0.38890600
Br	−2.45058300	2.36898900	−0.01258800
H	0.57893200	1.52940500	−0.84048000
H	−0.96125800	−3.15569200	−0.18217400
H	1.40806600	−2.69641200	−0.86614700
H	2.28363000	−0.25701000	1.37744100
H	4.23701400	0.13556300	2.84824400
H	6.50068500	0.51506300	1.87571100
H	6.77992600	0.49985300	−0.60670200
H	4.80744600	0.10428300	−2.07914500

neutral BDE-7, B3LYP/6-311G(d,p)

C	0.57439800	1.01889600	−0.02185400
C	1.89216500	0.57937900	0.01707800
C	2.15445200	−0.78540400	−0.04937100
C	1.11999700	−1.70573200	−0.15851900
C	−0.19602200	−1.25489300	−0.19986600
C	−0.48677400	0.10888600	−0.12943100
O	−1.75080200	0.62139500	−0.19005900
C	−2.85182100	−0.21939400	−0.06579700
C	−3.62627100	−0.46712200	−1.19355400
C	−4.78000500	−1.23924000	−1.06938300
C	−5.14734400	−1.75925000	0.16988200
C	−4.36158500	−1.49965000	1.29229500
C	−3.20946400	−0.72542200	1.18106200
Br	0.21496600	2.89081000	0.07060500
Br	3.97077700	−1.39388400	0.00966300
H	2.69722400	1.29556600	0.10111700
H	1.33056000	−2.76541900	−0.21510500
H	−1.00567600	−1.96702400	−0.29086500
H	−3.32535900	−0.04585100	−2.14492300
H	−5.39113300	−1.43203400	−1.94357000
H	−6.04537100	−2.35854200	0.26310600
H	−4.64919500	−1.89368000	2.26026900
H	−2.59598400	−0.50289300	2.04582000

anionic BDE-7, B3LYP/6-311G(d,p)

C	−0.26674500	0.28044300	−0.24232100
C	−1.60131000	0.47852700	0.08335600
C	−2.48633600	−0.58934500	−0.03224500
C	−2.07927600	−1.84646700	−0.46636700
C	−0.73528400	−2.03249000	−0.78022400
C	0.16271700	−0.96667000	−0.66489000
O	1.46505700	−1.25396900	−1.08283900
C	2.56285700	−1.08125100	−0.28388000
C	2.57279400	−0.41159400	0.94120700
C	3.76154200	−0.33584400	1.66368000
C	4.93852300	−0.90885400	1.18701800
C	4.92118400	−1.56437400	−0.04419100
C	3.74271900	−1.65288500	−0.77519500
Br	0.92847000	2.69762400	−0.33348700
Br	−4.36886700	−0.33370500	0.41720600
H	−1.94265800	1.45575900	0.40613900
H	−2.78712200	−2.66033300	−0.55427600
H	−0.37416900	−2.99860900	−1.12047700
H	1.68078500	0.08506500	1.29293000
H	3.76310400	0.20104600	2.60647500
H	5.85789300	−0.83343800	1.75770000
H	5.82868900	−2.01073100	−0.43882500
H	3.70661600	−2.15934600	−1.73277400

neutral BDE-7, B3LYP/6-31+G(d)

C	0.30481000	0.65519800	−0.23976700
C	1.65164000	0.66980900	0.12198400
C	2.43293200	−0.45739400	−0.12537900
C	1.89476300	−1.59306600	−0.73021900
C	0.54686700	−1.59357600	−1.08658000
C	−0.25321700	−0.47397900	−0.85203000
O	−1.54831600	−0.47544400	−1.31351000
C	−2.60461700	−0.68695900	−0.43831700
C	−3.87680800	−0.47008900	−0.97381700
C	−5.00087500	−0.71062100	−0.18537600
C	−4.86127700	−1.15051400	1.13619800
C	−3.58282800	−1.35694800	1.65732400
C	−2.44432600	−1.12148000	0.87856300
Br	−0.75510400	2.19966100	0.04715800

Br	4.27106500	−0.43101400	0.36192100
H	2.08110300	1.55064500	0.58448600
H	2.51499000	−2.46213600	−0.92115300
H	0.09899400	−2.45823300	−1.56706700
H	−3.96512400	−0.12555000	−1.99969300
H	−5.99050500	−0.54556600	−0.60364300
H	−5.73931100	−1.33440000	1.74889100
H	−3.46029300	−1.69964200	2.68163000
H	−1.45437300	−1.28649600	1.29137700

anionic BDE-7, B3LYP/6-31+G(d)

C	−0.30598600	0.40032200	−0.21607400
C	−1.63188500	0.58848800	0.15481100
C	−2.55389400	−0.43105100	−0.09245000
C	−2.18608000	−1.63845100	−0.68454800
C	−0.84415600	−1.82524400	−1.03185500
C	0.07346600	−0.79374000	−0.81002600
O	1.37895800	−1.02710500	−1.25449600
C	2.42949300	−1.01433500	−0.37531600
C	3.70478200	−0.98092800	−0.95415700
C	4.83999000	−1.06095400	−0.14988800
C	4.71899300	−1.15777900	1.24125200
C	3.44266000	−1.17647000	1.81060400
C	2.29620500	−1.09998800	1.01607900
Br	1.28664400	2.42061500	−0.15131800
Br	−4.41248100	−0.17881900	0.40059200
H	−1.94986500	1.52009700	0.61917700
H	−2.92042000	−2.41735200	−0.86249500
H	−0.51297500	−2.75893500	−1.48430600
H	3.78164000	−0.88582700	−2.03336200
H	5.82485300	−1.03015800	−0.61196500
H	5.60466000	−1.20706900	1.87050600
H	3.33034100	−1.23391200	2.89140300
H	1.31284700	−1.09211400	1.47180400

neutral BDE-7, B3LYP/6-31G(d)

C	−0.54652800	1.00126200	−0.05073500
C	−1.87821900	0.60477800	0.04341200
C	−2.19788200	−0.74307300	−0.10188900
C	−1.20729500	−1.69110800	−0.34633000
C	0.12112600	−1.28350200	−0.44650300
C	0.46765500	0.06353400	−0.29474300
O	1.74502300	0.53233000	−0.43744000
C	2.83330100	−0.28305900	−0.15748300
C	2.96095200	−0.93774900	1.06976300
C	4.11611800	−1.67636500	1.32655500
C	5.13616000	−1.75188200	0.37491100
C	4.99746400	−1.08293100	−0.84262500
C	3.84310000	−0.34789000	−1.11631300
Br	−0.10748100	2.84092900	0.15248600
Br	−4.02237900	−1.29115300	0.03608000
H	−2.64990200	1.34082100	0.23262100
H	−1.46792700	−2.73681700	−0.46569500
H	0.89854500	−2.01257600	−0.64724200
H	2.17045800	−0.86093800	1.80960500
H	4.22142200	−2.18624600	2.28025200
H	6.03462100	−2.32538100	0.58347300
H	5.78764200	−1.13341800	−1.58658500
H	3.71435200	0.18167400	−2.05482500

anionic BDE-7, B3LYP/6-31G(d)

C	−0.24320300	0.27421200	−0.32168800
C	−1.58010700	0.43987400	0.02535500
C	−2.43062700	−0.66261400	−0.01801200
C	−1.98590700	−1.92855400	−0.39528200
C	−0.64054700	−2.08408500	−0.72688800
C	0.22513200	−0.98140000	−0.68404500
O	1.52978000	−1.26042400	−1.10957800
C	2.63461300	−1.05672700	−0.31751500
C	3.68424000	−1.96250100	−0.52742200
C	4.86704700	−1.84679400	0.19972200
C	5.01087700	−0.83224200	1.14944100
C	3.96228700	0.06942700	1.34341500
C	2.77232800	−0.02530200	0.61836300
Br	0.69984700	2.80791700	−0.31852300
Br	−4.30691800	−0.45056300	0.45196500
H	−1.94684300	1.42228900	0.30841700
H	−2.66945500	−2.77041200	−0.42427500
H	−0.25056000	−3.05673500	−1.02094200
H	3.54725600	−2.75016400	−1.26308500
H	5.67366900	−2.55705000	0.02805600
H	5.93173300	−0.73901300	1.72071200
H	4.06895500	0.88033900	2.06048600
H	1.99769700	0.72697100	0.72410100

neutral BDE-5, B3LYP/6-311G(d,p)

C	0.79964900	−0.04003900	−0.02716300
C	1.81825400	0.91463500	−0.02103900
C	1.52205000	2.27731400	−0.05069400
C	0.19529100	2.68326200	−0.08912100
C	−0.83631000	1.75050100	−0.09817500
C	−0.54099600	0.38841600	−0.06643900

O	−1.49422600	−0.58971100	−0.08396600
C	−2.84240600	−0.25166400	−0.02377400
C	−3.44410200	−0.03559700	1.21211600
C	−4.81243400	0.22072300	1.26390700
C	−5.56631400	0.25710900	0.09180300
C	−4.94984300	0.03286000	−1.13784200
C	−3.58131000	−0.22173900	−1.20162100
Br	1.14863300	−1.90973100	0.01569900
Br	3.66135500	0.41408200	0.02923000
H	2.32656400	2.99965100	−0.04420900
H	−0.04062500	3.74052800	−0.11443400
H	−1.86856400	2.07142100	−0.13162000
H	−2.84433600	−0.08130300	2.11308100
H	−5.28981500	0.38536900	2.22305300
H	−6.63129500	0.45239000	0.13742600
H	−5.53378700	0.05195000	−2.05083700
H	−3.08308800	−0.40928800	−2.14496800

anionic BDE-5, B3LYP/6-311G(d,p)

C	0.50610000	0.24993800	−0.49831500
C	1.70137900	0.81625600	−0.15437200
C	1.82860200	2.19722200	−0.08452400
C	0.73236800	3.01179100	−0.37468200
C	−0.49018900	2.43603100	−0.72118300
C	−0.60775300	1.04927300	−0.78047200
O	−1.82876000	0.50789200	−1.19101800
C	−2.79796900	0.24375300	−0.26428700
C	−2.62660300	0.39164000	1.11431900
C	−3.68585100	0.09669200	1.97020700
C	−4.91134700	−0.34155200	1.47413700
C	−5.07057000	−0.48984700	0.09550300
C	−4.02411400	−0.20067600	−0.77124000
Br	0.30272600	−1.69574600	−0.63643300
Br	3.98709400	−0.29856300	0.63781200
H	2.78443900	2.63102200	0.19348500
H	0.82256100	4.09446700	−0.33263800
H	−1.35919300	3.04105600	−0.95440500
H	−1.67332600	0.72058400	1.50609300
H	−3.54180900	0.20722200	3.03991900
H	−5.72801800	−0.57005000	2.14979900
H	−6.01653400	−0.83534400	−0.30905900
H	−4.12606300	−0.31438400	−1.84388000

neutral BDE-5, B3LYP/6-31G(d)

C	0.67455300	0.24838400	−0.35752900
C	1.92479300	0.71149200	0.06878100
C	2.17214900	2.07963300	0.19131700
C	1.16718900	2.99489900	−0.11355700
C	−0.08263300	2.54898000	−0.53778200
C	−0.33024700	1.18243500	−0.65998900
O	−1.54029500	0.77354000	−1.16917700
C	−2.56093600	0.38328500	−0.31469200
C	−2.45374800	0.39237400	1.07621900
C	−3.55096800	−0.00917700	1.84229200
C	−4.73860500	−0.41320000	1.23358600
C	−4.82767000	−0.41592400	−0.16144100
C	−3.74329200	−0.01943900	−0.93986500
Br	0.29416000	−1.59497000	−0.54712700
Br	3.33186000	−0.49750500	0.49088400
H	3.14934600	2.41439200	0.52012700
H	1.36242100	4.05889300	−0.02077300
H	−0.87916500	3.24182600	−0.78799400
H	−1.53292400	0.70469000	1.55632600
H	−3.46783200	−0.00432000	2.92585000
H	−5.58589000	−0.72406000	1.83768100
H	−5.74684100	−0.72913900	−0.64920000
H	−3.79093900	−0.01641600	−2.02415800

anionic BDE-5, B3LYP/6-31G(d)

C	0.52510800	0.42749100	−0.50576400
C	1.76699300	0.86245200	−0.13204800
C	1.97880300	2.20972800	0.14577000
C	0.92274600	3.12000900	0.03540900
C	−0.34883600	2.67177900	−0.33655600
C	−0.55169900	1.31737500	−0.60777500
O	−1.81861100	0.92901500	−1.05546900
C	−2.70566400	0.35817800	−0.18353500
C	−2.41568200	0.06031300	1.15284200
C	−3.40621300	−0.50495000	1.95683600
C	−4.67858600	−0.77730700	1.45112500
C	−4.95558200	−0.47991700	0.11368600
C	−3.97848000	0.08477400	−0.70185000
Br	0.20438700	−1.45785000	−0.93384900
Br	3.76493900	−0.58608800	0.62724200
H	2.97036800	2.54394100	0.44788900
H	1.07820400	4.17922100	0.24100300
H	−1.19112300	3.35240700	−0.42564600
H	−1.42518900	0.25612600	1.54660400
H	−3.17135700	−0.73923800	2.99261400
H	−5.44130300	−1.21970700	2.08688200
H	−5.94020600	−0.68807500	−0.29975700
H	−4.17434600	0.32145800	−1.74345000

neutral BDE-5, B3LYP/6-31+G(d)

C	0.68146900	0.21020800	−0.35137400
C	1.92536800	0.71400500	0.04807700
C	2.14666000	2.09241100	0.10119800
C	1.12596400	2.97737500	−0.24234700
C	−0.11720900	2.49080200	−0.64392300
C	−0.33579400	1.11521600	−0.69691200
O	−1.53252800	0.65287000	−1.19379600
C	−2.57608100	0.34085200	−0.33525900
C	−2.53824800	0.55964300	1.04306400
C	−3.66217700	0.23520800	1.81117900
C	−4.80362400	−0.30535700	1.21685100
C	−4.82094200	−0.51842200	−0.16668800
C	−3.71097900	−0.19897600	−0.94691600
Br	0.33249800	−1.64237100	−0.46234000
Br	3.36016300	−0.43472600	0.51077100
H	3.11826800	2.46471600	0.40782700
H	1.30571300	4.04776700	−0.20204300
H	−0.92526600	3.15699100	−0.92913100
H	−1.65481000	0.97821100	1.51398900
H	−3.63467200	0.40518600	2.88452600
H	−5.67044200	−0.55636800	1.82161800
H	−5.70300400	−0.93937100	−0.64248900
H	−3.70513400	−0.35948200	−2.02071900

anionic BDE-5, B3LYP/6-31+G(d)

C	0.53102200	0.28304500	−0.40795000
C	1.74293100	0.80510700	−0.04091600
C	1.90466700	2.18496200	0.05197600
C	0.83476000	3.04278100	−0.23057400
C	−0.40184900	2.50885200	−0.60897200
C	−0.55104500	1.12423200	−0.69694500
O	−1.76904500	0.62626300	−1.16867700
C	−2.75727600	0.28321600	−0.28628500
C	−2.63649900	0.37956400	1.10560500
C	−3.72148900	0.02347900	1.91183600
C	−4.91997200	−0.42764300	1.35226400
C	−5.02656800	−0.52064100	−0.04099200
C	−3.95508300	−0.16788600	−0.85922500
Br	0.25657100	−1.63925800	−0.57764400
Br	3.92513000	−0.38868300	0.53274700
H	2.87299700	2.59115800	0.34075000
H	0.95848100	4.12377400	−0.16396600
H	−1.25024800	3.14475300	−0.84835100
H	−1.70551900	0.71757000	1.54750900
H	−3.61864000	0.09396600	2.99256600
H	−5.75596500	−0.70545700	1.98942900
H	−5.95046100	−0.87351100	−0.49486700
H	−4.01939000	−0.23881500	−1.94120200

neutral BDE-3, B3LYP/6-311G(d,p)

C	−0.53245500	1.67200700	0.58958000
C	−1.86924100	1.29018400	0.56400600
C	−2.22425800	0.08518900	−0.03515700
C	−1.26126100	−0.73398700	−0.61407300
C	0.07658100	−0.34708900	−0.59418900
C	0.44001500	0.85536800	0.01298400
O	1.72829600	1.34001900	0.02046000
C	2.81185700	0.47774400	0.03531000
C	2.90625200	−0.57478000	0.94548900
C	4.05796500	−1.35723000	0.96165000
C	5.10984000	−1.08942100	0.08679800
C	5.00617000	−0.02818900	−0.81012200
C	3.85609600	0.75661100	−0.84214600
Br	−4.06646300	−0.45068300	−0.06500500
H	−0.22793400	2.60398100	1.04936900
H	−2.62447700	1.92302300	1.01137000
H	−1.54763000	−1.66490500	−1.08589500
H	0.82794200	−0.97699400	−1.05262900
H	2.09258800	−0.77159900	1.63282600
H	4.13497700	−2.17491500	1.66935800
H	6.00440400	−1.70054500	0.10788400
H	5.82031200	0.19008100	−1.49179200
H	3.75429700	1.58718100	−1.52977400

anionic BDE-3, B3LYP/6-311G(d,p)

C	−0.05615200	−0.91215200	1.21341100
C	1.31434300	−0.62413600	1.20501700
C	1.98773300	−0.48441500	0.00059800
C	1.31517500	−0.62784600	−1.20384500
C	−0.05532300	−0.91586100	−1.21228600
C	−0.72372000	−1.05059900	0.00053800
O	−2.09105000	−1.39201100	0.00050000
C	−3.02781800	−0.40118300	0.00007000
C	−2.72935700	0.96528100	−0.00119200
C	−3.76861700	1.89387200	−0.00164700
C	−5.10023000	1.48549100	−0.00088600
C	−5.38909500	0.11979900	0.00035500
C	−4.36510500	−0.81888300	0.00084700
Br	4.57007600	0.51718200	−0.00026700
H	−0.60918200	−1.03330500	2.14075100

H	1.84715600	−0.50920000	2.14528400
H	1.84863000	−0.51578700	−2.14409400
H	−0.60774900	−1.03983700	−2.13961200
H	−1.69711200	1.28914200	−0.00178400
H	−3.52585900	2.95146000	−0.00261900
H	−5.90037500	2.21715400	−0.00125300
H	−6.42051900	−0.21811300	0.00096500
H	−4.57024200	−1.88300800	0.00181800

neutral BDE-3, B3LYP/6-31G(d)

C	−0.52508300	1.65561400	0.61396600
C	−1.86517600	1.27573200	0.59297100
C	−2.23038100	0.08720500	−0.03741900
C	−1.27449500	−0.71890000	−0.65212400
C	0.06631100	−0.33484300	−0.63803700
C	0.43963500	0.85162100	0.00113300
O	1.72975400	1.33468500	0.00215100
C	2.81237300	0.47135600	0.02754900
C	2.87274000	−0.62457700	0.89290200
C	4.02893400	−1.40495500	0.92145000
C	5.11849500	−1.09265200	0.10536300
C	5.04745700	0.01117300	−0.74688700
C	3.89425900	0.79472400	−0.79229200
Br	−4.06833500	−0.44262400	−0.05937800
H	−0.21292700	2.57485600	1.09897100
H	−2.61772200	1.89596200	1.06734700
H	−1.57246800	−1.63713300	−1.14659000
H	0.81411000	−0.95244400	−1.12353700
H	2.02924100	−0.85624400	1.53524200
H	4.07866300	−2.25698000	1.59414300
H	6.01624300	−1.70321800	0.13592300
H	5.89035200	0.26402900	−1.38429100
H	3.81778200	1.65655400	−1.44764400

anionic BDE-3, B3LYP/6-31G(d)

C	−0.03819100	−0.85769500	1.21625300
C	1.33063600	−0.55280100	1.20585200
C	2.01071500	−0.40594400	0.00112400
C	1.33185200	−0.55486000	−1.20403600
C	−0.03695700	−0.85977400	−1.21529700
C	−0.70511000	−1.00565800	0.00026600
O	−2.06496100	−1.37806500	−0.00016600
C	−3.02520700	−0.40851300	−0.00022500
C	−2.75875600	0.96723900	−0.00071800
C	−3.82205200	1.87179200	−0.00083100
C	−5.14622400	1.43048500	−0.00046200
C	−5.40260200	0.05583600	0.00002500
C	−4.35424400	−0.85978500	0.00014500
Br	4.56973700	0.47988400	−0.00034800
H	−0.59245100	−0.98743200	2.14476300
H	1.86118200	−0.43458200	2.15058100
H	1.86340100	−0.43820100	−2.14838700
H	−0.59029600	−0.99110400	−2.14413000
H	−1.73203000	1.31471600	−0.00099400
H	−3.60473300	2.93761100	−0.00122000
H	−5.96588400	2.14434200	−0.00054300
H	−6.42798100	−0.30802500	0.00032200
H	−4.53546200	−1.93067800	0.00052600

neutral BDE-3, B3LYP/6-31+G(d)

C	−0.53831500	1.66977300	0.59608500
C	−1.87982900	1.29190300	0.56097600
C	−2.23984200	0.08552500	−0.04078200
C	−1.27405700	−0.74276900	−0.61070800
C	0.06936600	−0.36276500	−0.58261500
C	0.43338100	0.84187400	0.02682800
O	1.72397800	1.32773700	0.02980200
C	2.81144300	0.46818500	0.03112500
C	2.91307500	−0.59464400	0.93402100
C	4.07760700	−1.36597700	0.94778600
C	5.13549400	−1.07355400	0.08075100
C	5.02404900	0.00129000	−0.80593900
C	3.85970500	0.77290200	−0.83850900
Br	−4.06935900	−0.44597200	−0.06690900
H	−0.23340700	2.60452500	1.05644700
H	−2.63716400	1.93039000	1.00383500
H	−1.56271200	−1.67663000	−1.08202800
H	0.82227600	−0.99818700	−1.03706600
H	2.09677000	−0.80853100	1.61739100
H	4.16015500	−2.19294600	1.64848800
H	6.04026900	−1.67475000	0.10103200
H	5.84253200	0.23995000	−1.48014100
H	3.75455900	1.61285500	−1.51865900

anionic BDE-3, B3LYP/6-31+G(d)

C	0.02621200	−0.77543200	−1.21678800
C	−1.33420100	−0.42671700	−1.20545600
C	−2.00603100	−0.25073900	0.00012400
C	−1.33373700	−0.42453600	1.20574500
C	0.02667800	−0.77329300	1.21715200
C	0.68816100	−0.93579600	0.00019800
O	2.03525100	−1.35437600	0.00030100

C	3.03168600	−0.41982600	0.00007200
C	2.81254300	0.96499500	−0.00094600
C	3.90817200	1.83368600	−0.00119400
C	5.21759900	1.34546200	−0.00045900
C	5.42564700	−0.03959000	0.00056500
C	4.34436400	−0.91896400	0.00083200
Br	−4.60173900	0.40997900	−0.00002500
H	0.57197800	−0.92534200	−2.14737800
H	−1.86303100	−0.29990100	−2.15008400
H	−1.86217200	−0.29602300	2.15036600
H	0.57282400	−0.92151100	2.14779700
H	1.79907100	1.35087500	−0.00143200
H	3.72769200	2.90652400	−0.00202600
H	6.06194100	2.03033600	−0.00066600
H	6.43781200	−0.43906500	0.00119700
H	4.49017200	−1.99565600	0.00161800

neutral BDE-2, B3LYP/6-311G(d,p)

C	1.03795500	−0.58657500	−0.09680000
C	2.14757400	0.24695400	−0.09120700
C	2.03831700	1.61757900	−0.30720900
C	0.77228600	2.14855900	−0.54225300
C	−0.36096400	1.33988900	−0.56243800
C	−0.22012000	−0.02949900	−0.33448500
O	−1.26504800	−0.92179600	−0.38944600
C	−2.55878400	−0.52093800	−0.09686600
C	−2.87169200	0.14387400	1.08825500
C	−4.20177400	0.44929500	1.36455200
C	−5.21176300	0.08733000	0.47414100
C	−4.88552000	−0.58650400	−0.70086300
C	−3.55773000	−0.88956700	−0.99315600
Br	3.87743700	−0.51804800	0.23243800
H	1.12176200	−1.65108700	0.07280800
H	2.91544100	2.24999000	−0.29548100
H	0.66810900	3.21269600	−0.72092000
H	−1.33708700	1.76363400	−0.75745400
H	−2.08493500	0.41016300	1.78349600
H	−4.44877100	0.96429000	2.28601500
H	−6.24519400	0.32424900	0.69790400
H	−5.66463000	−0.87590400	−1.39681700
H	−3.28130300	−1.41437000	−1.89935600

anionic BDE-2, B3LYP/6-311G(d,p)

C	0.51697000	1.19048700	−0.46281600
C	0.38564300	2.39365100	0.22436300
C	−0.55692400	0.31302700	−0.61487900
C	−0.85582900	2.72101800	0.77391700
C	−1.77885500	0.65673000	−0.05712600
C	4.62318200	−1.52113300	0.73285300
C	−1.93936500	1.84774000	0.63671300
C	2.65109800	0.09826000	−0.42570100
C	3.41843300	−1.28793100	1.39189700
C	2.43054300	−0.48373700	0.82679100
C	3.85961300	−0.13396700	−1.09531100
C	4.83445600	−0.93697100	−0.51714500
O	1.75823800	0.90478600	−1.06657800
H	1.24107500	3.05377700	0.31276100
H	−0.97127400	3.66094100	1.30968600
H	5.38334800	−2.15052600	1.18187200
H	−2.90424700	2.09971800	1.06820600
H	3.23445300	−1.73924200	2.36136400
H	1.49330200	−0.31538900	1.34002200
H	4.00512100	0.32575300	−2.06582000
H	5.76533500	−1.10923800	−1.04804900
Br	−3.89776300	−1.13120000	−0.09269800
H	−0.42510400	−0.61512300	−1.16431500

neutral BDE-2, B3LYP/6-31G(d)

C	1.03646600	−0.58942200	−0.10079900
C	2.15092000	0.24214300	−0.09334200
C	2.04732600	1.61370800	−0.32000900
C	0.78286000	2.14957200	−0.56746500
C	−0.35474900	1.34269500	−0.59047300
C	−0.21980100	−0.02853000	−0.35141100
O	−1.26887400	−0.91743300	−0.41254400
C	−2.55899500	−0.51578200	−0.10456700
C	−2.85644900	0.18324000	1.06841100
C	−4.18657600	0.48683300	1.35975900
C	−5.21145500	0.08888500	0.49806800
C	−4.89985900	−0.61852800	−0.66461800
C	−3.57294100	−0.92037600	−0.97292400
Br	3.87227800	−0.52188200	0.24507500
H	1.12064500	−1.65456200	0.07854600
H	2.93034800	2.24188300	−0.30549300
H	0.68328300	3.21527200	−0.75421800
H	−1.33105400	1.76722500	−0.79509000
H	−2.05732200	0.47804400	1.74107200
H	−4.42112900	1.02965900	2.27145100
H	−6.24503700	0.32558500	0.73341000
H	−5.69038200	−0.93520100	−1.33948400
H	−3.30858300	−1.46919000	−1.87123800

anionic BDE-2, B3LYP/6-31G(d)

C	0.58119800	0.15225600	−0.54986600
C	1.78673000	0.52101700	0.03746600
C	1.92198500	1.76066900	0.65542800
C	0.84048100	2.65013300	0.68936100
C	−0.38093700	2.29524700	0.10626500
C	−0.49187900	1.04628100	−0.50544500
O	−1.70819600	0.72603500	−1.14636400
C	−2.66997300	0.04526000	−0.45988000
C	−2.52912900	−0.40583900	0.85957800
C	−3.58294900	−1.09040900	1.46701100
C	−4.77609200	−1.33357300	0.78439900
C	−4.90715300	−0.88151200	−0.53227900
C	−3.86602200	−0.19758500	−1.15332500
Br	3.98585100	−1.04463200	−0.07027500
H	0.45965400	−0.81147700	−1.04312300
H	2.87214800	2.03802000	1.11075100
H	0.93956100	3.62602400	1.16689500
H	−1.23514600	2.96698400	0.11226900
H	−1.60173400	−0.22663600	1.39120900
H	−3.46172800	−1.43943100	2.49013600
H	−5.58942000	−1.86872000	1.26768100
H	−5.82850100	−1.06227400	−1.08214700
H	−3.95159200	0.15966200	−2.17541000

neutral BDE-2, B3LYP/6-31+G(d)

C	1.03763200	−0.58958900	−0.13359300
C	2.15235400	0.24278100	−0.09205500
C	2.04616500	1.62282500	−0.26820600
C	0.78179500	2.16839600	−0.50175000
C	−0.35578700	1.36116800	−0.55895600
C	−0.21697400	−0.01713100	−0.36557100
O	−1.26877800	−0.90180800	−0.47005700
C	−2.55641300	−0.50931200	−0.13423400
C	−2.84319700	0.08573500	1.09790800
C	−4.17048100	0.37960500	1.41831500
C	−5.20263900	0.06944500	0.52643900
C	−4.90063400	−0.53911900	−0.69508100
C	−3.57547200	−0.82747900	−1.03260500
Br	3.86462900	−0.52721700	0.23532200
H	1.11981700	−1.66147400	0.00558900
H	2.92685700	2.25401900	−0.22639900
H	0.68294000	3.24050000	−0.65031300
H	−1.33188200	1.79229900	−0.75328200
H	−2.03953000	0.30943700	1.79308500
H	−4.39775200	0.84245100	2.37524700
H	−6.23348700	0.29561200	0.78469600
H	−5.69634700	−0.78843900	−1.39225800
H	−3.32049500	−1.30128300	−1.97583500

anionic BDE-2, B3LYP/6-31+G(d)

C	0.59575800	0.15246800	−0.56985300
C	1.80939300	0.52521700	−0.00110300
C	1.94704900	1.75953300	0.62777000
C	0.85915700	2.64269100	0.68890600
C	−0.37029800	2.28631700	0.12017300
C	−0.48123100	1.04065800	−0.49875500
O	−1.69714800	0.72072000	−1.13948600
C	−2.66842900	0.04669100	−0.45600800
C	−2.53861400	−0.39169400	0.86925900
C	−3.59983600	−1.07107300	1.47495900
C	−4.78844300	−1.31902500	0.78299300
C	−4.90759600	−0.87750500	−0.54081000
C	−3.85882300	−0.19951800	−1.15947500
Br	3.96989900	−1.05011400	−0.06051200
H	0.47327100	−0.80612300	−1.07284600
H	2.90300700	2.04190600	1.06760300
H	0.96367900	3.61437000	1.17368000
H	−1.22677700	2.95524000	0.14423900
H	−1.61716200	−0.20838900	1.41084900
H	−3.48721400	−1.41132400	2.50215000
H	−5.60662800	−1.84968800	1.26359200
H	−5.82405400	−1.06317500	−1.09741000
H	−3.93592600	0.14685300	−2.18638700

neutral BDE-1 B3LYP/6-311G(d,p)

C	1.75793800	0.25528000	−0.02172100
C	2.71615400	1.25522600	0.10476100
C	2.34536300	2.59305800	−0.00374900
C	1.01477800	2.92173700	−0.24829700
C	0.05587300	1.92302000	−0.38215100
C	0.41710100	0.57857400	−0.26467700
O	−0.46647500	−0.45207500	−0.44013400
C	−1.81469000	−0.28536200	−0.16458400
C	−2.25608300	0.17040500	1.07677000
C	−3.62400600	0.24191900	1.32537600
C	−4.54237300	−0.14583400	0.35038700
C	−4.08591700	−0.60987900	−0.88138700
C	−2.71983400	−0.67833200	−1.14554600
Br	2.28039800	−1.57675900	0.13266500
H	3.74616000	0.98075000	0.29283200
H	3.09485600	3.36831800	0.09996600

H	0.71722300	3.95949200	−0.34343600
H	−0.97760200	2.17546700	−0.58264300
H	−1.53664800	0.45600600	1.83446900
H	−3.97175700	0.59334500	2.29019800
H	−5.60560400	−0.09195200	0.55252300
H	−4.79293600	−0.91821800	−1.64305700
H	−2.34165200	−1.03891400	−2.09416300

anionic BDE-1 B3LYP/6-311G(d,p)

C	0.66743000	−1.46476500	−0.30306400
C	1.21638700	−2.74537600	−0.15269300
C	1.41753400	−0.34307800	0.00514300
C	2.51968100	−2.89205000	0.31291300
C	2.71244700	−0.49027500	0.48018100
C	−4.02926800	0.36867700	0.69233600
C	3.27204800	−1.76169700	0.63620600
C	−1.68504000	−0.79907600	−0.29577100
C	−2.77728100	0.82412900	1.10053300
C	−1.60075800	0.25540600	0.61571700
C	−2.93885400	−1.25767500	−0.72039200
C	−4.10169100	−0.67488500	−0.23074100
O	−0.61959800	−1.45653200	−0.85193500
Br	1.20297400	2.47030900	−0.27167800
H	0.60807900	−3.60734800	−0.41023300
H	2.94231100	−3.88584700	0.42563800
H	3.28719700	0.39997000	0.71436100
H	−4.93434600	0.82917100	1.07437600
H	4.29015400	−1.86942300	1.00140400
H	−2.70049200	1.65567300	1.79318800
H	−0.63816400	0.66714000	0.88653300
H	−2.97152500	−2.07321400	−1.43389400
H	−5.06633300	−1.04067300	−0.56936800

neutral BDE-1 B3LYP/6-31G(d)

C	−1.58625500	0.18897900	−0.00055200
C	−2.83841500	−0.06225200	0.55907800
C	−3.34606500	−1.36169700	0.56724400
C	−2.60280200	−2.40703600	0.01637400
C	−1.34998800	−2.15276900	−0.53983100
C	−0.83332100	−0.85557300	−0.55121300
O	0.36440600	−0.62123900	−1.18865000
C	1.53452100	−0.51813500	−0.45386800
C	1.60273800	−0.70319200	0.92760400
C	2.84053700	−0.59214400	1.56593100
C	3.99606100	−0.30285200	0.84107100
C	3.90944200	−0.12134200	−0.54228900
C	2.68346900	−0.22733100	−1.19385300
Br	−0.89838700	1.96233900	−0.02233000
H	−3.40830000	0.75979900	0.97871400
H	−4.32362800	−1.55130800	1.00075700
H	−2.99637500	−3.41924100	0.01764900
H	−0.75426500	−2.94623700	−0.98018200
H	0.70807400	−0.92842600	1.49751900
H	2.89358000	−0.73412600	2.64215500
H	4.95359200	−0.21807200	1.34653500
H	4.80155700	0.10529500	−1.12004600
H	2.59453100	−0.08758300	−2.26652400

anionic BDE-1 B3LYP/6-31G(d)

C	1.34486000	0.43818700	0.00543200
C	2.61208000	0.69562100	0.51783900
C	3.06512100	2.01032600	0.67848100
C	2.23328400	3.07714200	0.32334000
C	0.95640000	2.82273000	−0.17542700
C	0.51423200	1.49856300	−0.32695100
O	−0.76286800	1.39128400	−0.89503300
C	−1.78172400	0.65875800	−0.33588500
C	−1.62148000	−0.55558200	0.34142300
C	−2.75614800	−1.20189600	0.83757000
C	−4.03630900	−0.67125500	0.66416900
C	−4.18398600	0.53273100	−0.02942200
C	−3.06354300	1.19699300	−0.52391800
Br	1.50678900	−2.36233600	−0.23235300
H	3.24868700	−0.14770200	0.77372400
H	4.06296600	2.20291700	1.07189700
H	2.57266100	4.10505400	0.43887700
H	0.28546800	3.63416900	−0.45136200
H	−0.64118500	−1.01575900	0.42931600
H	−2.62524700	−2.15045200	1.35363500
H	−4.90792100	−1.19278400	1.05372100
H	−5.17266800	0.96253300	−0.18031000
H	−3.15415300	2.13958200	−1.05679100

neutral BDE-1 B3LYP/6-31+G(d)

C	−1.57642500	0.20892900	0.02015900
C	−2.83866900	−0.03354700	0.56388400
C	−3.37416800	−1.32317200	0.54227200
C	−2.65188100	−2.37154600	−0.03405900
C	−1.39243900	−2.12846600	−0.58349000
C	−0.85368600	−0.84013100	−0.56141800
O	0.34728200	−0.61199400	−1.19704200
C	1.52055600	−0.52271100	−0.46412200

C	1.61418400	−0.84476700	0.89140600
C	2.86097000	−0.76798100	1.52189100
C	3.99566800	−0.36196200	0.81738100
C	3.88046600	−0.03747500	−0.53955700
C	2.64572600	−0.10814200	−1.18250500
Br	−0.85855000	1.96450200	0.02159600
H	−3.39816700	0.78744800	1.00096300
H	−4.35765000	−1.50110700	0.96807000
H	−3.06679100	−3.37534400	−0.05809300
H	−0.81351200	−2.92195500	−1.04667000
H	0.73786700	−1.16297900	1.44658100
H	2.93614500	−1.02333200	2.57593700
H	4.95907100	−0.30286800	1.31597200
H	4.75562300	0.28117200	−1.10018300
H	2.53659700	0.14318200	−2.23314600

anionic BDE-1 B3LYP/6-31+G(d)

C	−1.46890100	−0.25816300	0.04970800
C	−2.69645400	−0.52968600	0.64762900
C	−3.17082000	−1.84425500	0.76245600
C	−2.38842600	−2.90510700	0.28917100
C	−1.14145000	−2.64264300	−0.28432300
C	−0.69197900	−1.31782200	−0.39209800
O	0.54947300	−1.17047600	−1.03450300
C	1.64179100	−0.66036600	−0.37888100
C	1.58020600	0.25365400	0.68101800
C	2.76807100	0.66650000	1.29002400
C	4.01407800	0.23374600	0.82540300
C	4.06545600	−0.64914600	−0.25928700
C	2.88681800	−1.09706700	−0.85710500
Br	−1.20822400	2.37665800	−0.28578100
H	−3.30399700	0.30111600	1.00466300
H	−4.14136000	−2.04039700	1.21821300
H	−2.74174100	−3.93172800	0.36956500
H	−0.50898100	−3.44979100	−0.65062200
H	0.62452700	0.64129700	1.01053400
H	2.71138400	1.37172300	2.11657100
H	4.93050600	0.58008300	1.29851700
H	5.02458100	−0.99617600	−0.63980700
H	2.90679000	−1.79320600	−1.69154700

neutral BDE-21 B3LYP/6-31G(d,p)

C	−0.09001500	0.70185200	−0.09977400
C	−1.35150300	0.09207800	−0.03958600
C	−1.44545800	−1.30404200	−0.12406500
C	−0.29417300	−2.07304000	−0.27379100
C	0.95578300	−1.46993200	−0.33989500
C	1.06754600	−0.08128100	−0.24837600
O	2.25751900	0.58296400	−0.34768400
C	3.45426500	−0.08149200	−0.10347100
C	3.73059000	−0.62512500	1.15244600
C	4.97661800	−1.21003100	1.37689800
C	5.93679000	−1.24148000	0.36263800
C	5.64784300	−0.68406800	−0.88388200
C	4.40178100	−0.10296000	−1.12458800
Br	0.11258100	2.58314600	0.01181400
Br	−2.91171800	1.14626300	0.15964300
Br	−3.11799900	−2.20633000	−0.04033400
H	−0.38205600	−3.15046800	−0.34491800
H	1.84686500	−2.07300500	−0.46466600
H	2.98175800	−0.58086500	1.93630600
H	5.19963200	−1.63346800	2.35158200
H	6.90627000	−1.69417900	0.54541800
H	6.39144200	−0.70141500	−1.67485900
H	4.15530500	0.33903800	−2.08399000

anionic BDE-21 B3LYP/6-31G(d,p)

C	0.11459800	0.09199100	−0.60526000
C	−1.21942000	−0.01400900	−0.30521500
C	−1.76414500	−1.26584500	−0.18161200
C	−1.01288700	−2.43154900	−0.33547700
C	0.34855900	−2.31130000	−0.61582500
C	0.91822000	−1.04372700	−0.75877400
O	2.25886000	−0.97782600	−1.14121600
C	3.22843300	−0.75219700	−0.19973200
C	4.54945900	−0.83325100	−0.65817700
C	5.60546800	−0.63607600	0.22725400
C	5.35907900	−0.35762500	1.57431000
C	4.03887900	−0.27426700	2.01952800
C	2.96893200	−0.46832800	1.14534200
Br	0.96409900	1.83931100	−0.87017400
Br	−2.41435400	1.91960300	0.60390600
Br	−3.68382700	−1.51356600	0.18892700
H	−1.47625700	−3.40937000	−0.24107000
H	0.97970200	−3.18680600	−0.73371500
H	4.72023200	−1.05018400	−1.70776100
H	6.62717600	−0.70197100	−0.13861100
H	6.18353000	−0.20304300	2.26445900
H	3.83026600	−0.04889700	3.06208700
H	1.94629000	−0.38720300	1.49313200

neutral BDE-12 B3LYP/6-31G(d,p)

C	−0.05778200	1.20893600	−0.12923800
C	−1.31388800	0.61189200	−0.04980900
C	−1.44270800	−0.77706800	−0.15505000
C	−0.29985400	−1.55435300	−0.34789500
C	0.95981400	−0.96920000	−0.43533900
C	1.07766300	0.41926300	−0.32096700
O	2.26649600	1.09590500	−0.44874300
C	3.46617100	0.47183600	−0.13493100
C	3.67297700	−0.13062400	1.10835900
C	4.92413000	−0.67412000	1.39893400
C	5.96025800	−0.60808300	0.46461300
C	5.74112000	0.00680000	−0.76917900
C	4.49150100	0.54645500	−1.07646900
Br	−3.12980100	−1.64721000	−0.04286700
Br	−2.82511600	1.73573600	0.21370500
H	0.04947600	2.28339000	−0.04590400
H	−0.40351700	−2.62961700	−0.43664700
H	1.83729500	−1.58464000	−0.59305000
H	2.86667400	−0.16576400	1.83333400
H	5.09031700	−1.14276400	2.36434600
H	6.93257500	−1.02992700	0.69886400
H	6.54246300	0.06477400	−1.49963300
H	4.29841300	1.02849500	−2.02885800

anionic BDE-12 B3LYP/6-31G(d,p)

C	0.24470700	0.52783900	−0.67171500
C	−1.06157400	0.24230600	−0.28588000
C	−1.44485000	−1.06397600	−0.14666500
C	−0.57788500	−2.13512600	−0.37730900
C	0.73551200	−1.85676100	−0.76092700
C	1.13219800	−0.52449000	−0.90148600
O	2.43824000	−0.26234200	−1.35486000
C	3.44041100	−0.07252400	−0.44674200
C	3.25869300	−0.08553500	0.94203000
C	4.35810800	0.11574700	1.77801500
C	5.63401000	0.32856700	1.25462600
C	5.80453200	0.34198700	−0.13289800
C	4.71947000	0.14347400	−0.98123400
Br	−3.28129600	−1.52024600	0.40165200
Br	−2.50875400	2.36535600	−0.00914800
H	0.57400400	1.55751600	−0.79899100
H	−0.91527800	−3.16208900	−0.26313500
H	1.44566600	−2.65402500	−0.95826100
H	2.26866800	−0.24240300	1.35297600
H	4.20732000	0.10757400	2.85447100
H	6.48177800	0.48466300	1.91543400
H	6.79064900	0.50846700	−0.55949900
H	4.83303400	0.15110800	−2.06063800

neutral BDE-7 B3LYP/6-31G(d,p)

C	−0.54742600	1.00179900	−0.04970400
C	−1.87870500	0.60423800	0.04292600
C	−2.19650300	−0.74396300	−0.10133700
C	−1.20490900	−1.69130900	−0.34314700
C	0.12304900	−1.28270400	−0.44162200
C	0.46798200	0.06470700	−0.29081100
O	1.74509900	0.53463900	−0.43146300
C	2.83351100	−0.28178200	−0.15531400
C	2.96314200	−0.93856900	1.07050000
C	4.11833300	−1.67795800	1.32342000
C	5.13633400	−1.75190100	0.36986800
C	4.99567500	−1.08077700	−0.84590900
C	3.84129300	−0.34495800	−1.11610300
Br	−0.11068400	2.84193400	0.15203000
Br	−4.02035300	−1.29357200	0.03447100
H	−2.65093700	1.33945700	0.23002000
H	−1.46438900	−2.73682000	−0.46154800
H	0.90139900	−2.01073700	−0.63949200
H	2.17429000	−0.86329100	1.81136000
H	4.22545600	−2.18956600	2.27526300
H	6.03441700	−2.32579000	0.57551900
H	5.78412600	−1.13024200	−1.59083700
H	3.71051800	0.18626600	−2.05271200

anionic BDE-7 B3LYP/6-31G(d,p)

C	0.25346800	0.24680100	−0.33756400
C	1.59039100	0.45141100	−0.01335800
C	2.46024200	−0.63664300	−0.01518900
C	2.03418600	−1.92689400	−0.32478700
C	0.68854800	−2.12218200	−0.63264500
C	−0.19514800	−1.03327100	−0.63367500
O	−1.49561900	−1.35306000	−1.03808200
C	−2.61065800	−1.08798800	−0.28266400
C	−3.72057700	−1.89127000	−0.58304700
C	−4.91638700	−1.72368100	0.11035300
C	−5.01645900	−0.75884700	1.11577900
C	−3.90801800	0.04060800	1.39990800
C	−2.70179800	−0.10832600	0.71253600
Br	−0.78243200	2.73472100	−0.33675800
Br	4.33735600	−0.36961800	0.42155700
H	1.94123500	1.45158600	0.22178900
H	2.73200800	−2.75656900	−0.32053200

H	0.31274900	−3.11376300	−0.87525700
H	−3.61800300	−2.64124000	−1.36151200
H	−5.76884500	−2.35408300	−0.13240700
H	−5.94792800	−0.62402900	1.65931000
H	−3.97764200	0.81364800	2.16091400
H	−1.87771300	0.57200800	0.89051300

neutral BDE-5 B3LYP/6-31G(d,p)

C	0.67393600	0.24894200	−0.35775200
C	1.92428600	0.71104700	0.06925200
C	2.17223500	2.07879100	0.19318700
C	1.16802500	2.99469100	−0.11084500
C	−0.08176300	2.55011300	−0.53565200
C	−0.33014700	1.18402900	−0.65934000
O	−1.54051300	0.77649200	−1.16903500
C	−2.56011500	0.38417200	−0.31447100
C	−2.45041800	0.38911600	1.07614900
C	−3.54656800	−0.01375100	1.84252800
C	−4.73514700	−0.41530300	1.23463100
C	−4.82653200	−0.41393900	−0.15997000
C	−3.74350000	−0.01595100	−0.93903100
Br	0.29260000	−1.59390700	−0.54904400
Br	3.33067100	−0.49873600	0.49046700
H	3.14910700	2.41221100	0.52240100
H	1.36369700	4.05787600	−0.01696400
H	−0.87774300	3.24296700	−0.78511400
H	−1.52904700	0.69951500	1.55505400
H	−3.46209300	−0.01207200	2.92534800
H	−5.58111700	−0.72711400	1.83884500
H	−5.74626300	−0.72503900	−0.64659000
H	−3.79266500	−0.00951100	−2.02262600

anionic BDE-5 B3LYP/6-31G(d,p)

C	0.52471300	0.42846400	−0.50753000
C	1.76720900	0.86207900	−0.13398200
C	1.97984900	2.20877400	0.14568900
C	0.92444600	3.11960100	0.03719800
C	−0.34757300	2.67296500	−0.33418700
C	−0.55154500	1.31924000	−0.60710700
O	−1.81914200	0.93234300	−1.05416300
C	−2.70444500	0.35914200	−0.18219400
C	−2.41152200	0.05860100	1.15285400
C	−3.40027700	−0.50836400	1.95728800
C	−4.67327400	−0.78012200	1.45347800
C	−4.95295700	−0.48018300	0.11745300
C	−3.97792800	0.08638900	−0.69882100

Br	0.20263600	−1.45583400	−0.93845400
Br	3.76200000	−0.58802100	0.62903300
H	2.97118300	2.54192800	0.44740200
H	1.08073300	4.17775400	0.24390000
H	−1.18928800	3.35347700	−0.42153100
H	−1.42066400	0.25408400	1.54434700
H	−3.16383500	−0.74449300	2.99158800
H	−5.43413000	−1.22360900	2.08944100
H	−5.93777400	−0.68775800	−0.29399900
H	−4.17550900	0.32526800	−1.73893100

neutral BDE-3 B3LYP/6-31G(d,p)

C	−0.52616900	1.65734900	0.61161000
C	−1.86595600	1.27746100	0.58983300
C	−2.22994200	0.08701300	−0.03719400
C	−1.27342300	−0.72114100	−0.64774000
C	0.06713600	−0.33720700	−0.63296900
C	0.43920500	0.85126200	0.00294900
O	1.72920700	1.33452800	0.00468700
C	2.81188700	0.47135100	0.02837600
C	2.87378500	−0.62400600	0.89422500
C	4.02997000	−1.40395800	0.92087800
C	5.11784700	−1.09179900	0.10294200
C	5.04527300	0.01142600	−0.74946900
C	3.89212300	0.79468600	−0.79333300
Br	−4.06754800	−0.44278000	−0.06015200
H	−0.21427900	2.57756000	1.09364300
H	−2.61900800	1.89872600	1.06071000
H	−1.57081200	−1.64046700	−1.13924600
H	0.81544700	−0.95622300	−1.11454800
H	2.03170500	−0.85562800	1.53741200
H	4.08125700	−2.25528500	1.59330000
H	6.01516200	−1.70187000	0.13218100
H	5.88669500	0.26381500	−1.38790600
H	3.81393300	1.65584300	−1.44837300

anionic BDE-3 B3LYP/6-31G(d,p)

C	0.70499900	−1.00768900	0.00000000
O	2.06507900	−1.37946100	−0.00000400
C	3.02393800	−0.40876600	−0.00000200
C	2.75476100	0.96636400	0.00000100
C	3.81629200	1.87249800	0.00000300
C	5.14102300	1.43359200	0.00000200
C	5.39991500	0.05968500	0.00000000
C	4.35357100	−0.85784900	−0.00000300
C	0.03762000	−0.86124200	−1.21573200

C	−1.33111600	−0.55705100	−1.20489500
C	−2.01058000	−0.40990700	0.00000400
C	−1.33111300	−0.55705700	1.20490000
C	0.03762300	−0.86124800	1.21573200
Br	−4.56589600	0.48180500	0.00000000
H	1.72757800	1.31089900	0.00000100
H	3.59748800	2.93733800	0.00000500
H	5.95881000	2.14847000	0.00000300
H	6.42539600	−0.30182900	0.00000000
H	4.53621700	−1.92785500	−0.00000500
H	0.59155200	−0.99131500	−2.14362200
H	−1.86223400	−0.43992000	−2.14866400
H	−1.86222800	−0.43993000	2.14867100
H	0.59155800	−0.99132600	2.14362000

neutral BDE-2 B3LYP/6-31G(d,p)

C	1.03335000	−0.58775500	−0.11072100
C	2.14929000	0.24163000	−0.09409400
C	2.04986700	1.61400200	−0.31617600
C	0.78814400	2.15351300	−0.56868300
C	−0.35067800	1.34936200	−0.60141200
C	−0.21973900	−0.02293700	−0.36662400
O	−1.27107500	−0.90832200	−0.43992700
Br	3.86690400	−0.52683300	0.25104300
C	−2.55813000	−0.51123200	−0.11477200
C	−2.84421200	0.18921500	1.06009600
C	−4.17188200	0.48786200	1.36630800
C	−5.20525100	0.08384500	0.51824900
C	−4.90474600	−0.62492700	−0.64623000
C	−3.58079800	−0.92210400	−0.96977500
H	1.11381700	−1.65322900	0.06496100
H	2.93367300	2.23980000	−0.29437400
H	0.69212400	3.21954200	−0.75180900
H	−1.32474400	1.77609100	−0.80917000
H	−2.03904800	0.48957800	1.72196300
H	−4.39770400	1.03164600	2.27888200
H	−6.23622200	0.31689800	0.76527400
H	−5.70165000	−0.94605300	−1.31038000
H	−3.32460400	−1.47138700	−1.86944800

anionic BDE-2 B3LYP/6-31G(d,p)

C	0.54609400	0.23838700	−0.63754100
C	1.78125100	0.54939400	−0.07960500
C	1.95507000	1.72869300	0.63897400
C	0.88519100	2.61715700	0.79962600
C	−0.36556400	2.32055100	0.24693900
C	−0.51610700	1.13056600	−0.46445500
O	−1.76232300	0.87689600	−1.07587900
C	−2.68139800	0.10127000	−0.43239600
C	−2.47869900	−0.48431100	0.82447300
C	−3.49398300	−1.25440600	1.39340400
C	−4.70831100	−1.45079000	0.73418700
C	−4.90082800	−0.86408600	−0.52011200
C	−3.89884900	−0.09396000	−1.10283600
Br	3.95585900	−1.06641400	−0.08943400
H	0.39109100	−0.67549000	−1.20903000
H	2.92731200	1.95702000	1.07208800
H	1.01547400	3.54638500	1.35472300
H	−1.21243800	2.99224100	0.35194600
H	−1.53460900	−0.34172600	1.33633100
H	−3.32553300	−1.70760800	2.36706900
H	−5.49057800	−2.05315300	1.18705000
H	−5.83906200	−1.00718300	−1.05079200
H	−4.03133500	0.36806100	−2.07609400

neutral BDE-1 B3LYP/6-31G(d,p)

C	1.58455700	0.19101600	−0.00111000
C	2.83721300	−0.05451400	0.55946700
C	3.34937800	−1.35187200	0.57037200
C	2.61036100	−2.40083500	0.02145100
C	1.35703900	−2.15250200	−0.53561200
C	0.83563100	−0.85745300	−0.54987300
O	−0.36283000	−0.62954300	−1.18829200
C	−1.53226500	−0.52277600	−0.45313400
C	−1.59811300	−0.69740700	0.92968800
C	−2.83517200	−0.58418500	1.56843100
C	−3.99191900	−0.30274000	0.84296000
C	−3.90749400	−0.13149500	−0.54157700
C	−2.68246300	−0.23988200	−1.19394700
Br	0.89066200	1.96173900	−0.02674000
H	3.40357400	0.77005200	0.97741900
H	4.32683300	−1.53711900	1.00444700
H	3.00750900	−3.41095500	0.02508000
H	0.76425600	−2.94835800	−0.97422600
H	−0.70258300	−0.91631900	1.49949700
H	−2.88688800	−0.71815000	2.64510500
H	−4.94838600	−0.21626100	1.34867700
H	−4.80043500	0.08880100	−1.11930000
H	−2.59491200	−0.10835300	−2.26716000

anionic BDE-1 B3LYP/6-31G(d,p)

C	−1.35807400	0.41580400	−0.00412700
C	−2.63792100	0.63895400	0.49246500

C	−3.12402500	1.94089000	0.65809100
C	−2.31230500	3.02990900	0.32547200
C	−1.02293900	2.81045500	−0.15663100
C	−0.54781900	1.49849900	−0.31444600
O	0.73775500	1.42809600	−0.86725500
C	1.76466600	0.69851700	−0.32075500
C	1.61763900	−0.50492400	0.37849500
C	2.76209000	−1.14332400	0.86162500
C	4.03838300	−0.61605100	0.65548000
C	4.17218200	0.57689200	−0.05932400
C	3.04272500	1.23340200	−0.54215200
Br	−1.43758400	−2.38459800	−0.23714800
H	−3.25680500	−0.22158500	0.73196700
H	−4.13088500	2.10650300	1.03856900
H	−2.67708500	4.04775700	0.44563200
H	−0.36743900	3.63916200	−0.41526600
H	0.64043600	−0.96530000	0.49048600
H	2.64144500	−2.08371200	1.39351600
H	4.91682200	−1.13156400	1.03544200
H	5.15703000	1.00377700	−0.23619200
H	3.12226200	2.16698900	−1.09112200
